# Four undescribed pyrethrins from seeds of *Pyrethrum* *cinerariifolium* and their aphidicidal activity

**DOI:** 10.1007/s13659-023-00385-0

**Published:** 2023-07-07

**Authors:** Hao-Ran Zhou, Li-Wu Lin, Zhong-Rong Li, Xing-Rong Peng, Ming-Hua Qiu

**Affiliations:** 1 State Key Laboratory of Phytochemistry and Plant Resources in West China, Kunming Institute of Botany, Chinese Academy of Sciences, Kunming, 650201 People’s Republic of China; 2 University of Chinese Academy of Sciences, Beijing, 100049 People’s Republic of China

**Keywords:** *Pyrethrumcinerariifolium*, Pyrethrins, Aphidicidal activity

## Abstract

Four undescribed pyrethrins C-F (**1**–**4**) as well as four known pyrethrins (**5**–**8**) were isolated from seeds of *Pyrethrum* *cinerariifolium* Trev. The structures of compounds **1**–**4** were elucidated by UV, HRESIMS, and NMR (^1^H and ^13^C NMR, ^1^H-^1^H COSY, HSQC, HMBC and ROESY), among which the stereostructure of compound **4** was determined by calculated ECD. Furthermore, compounds **1**–**4** were evaluated for their aphidicidal activities. The insecticidal assay results showed that **1**–**4** exhibited moderate aphidicidal activities at the concentration of 0.1 mg/mL with the 24 h mortality rates ranging from 10.58 to 52.98%. Among them, pyrethrin D (**2**) showed the highest aphidicidal activity, with the 24 h mortality rate of 52.98%, which was slightly lower than the positive control (pyrethrin II, 83.52%).

**Graphical Abstract:**

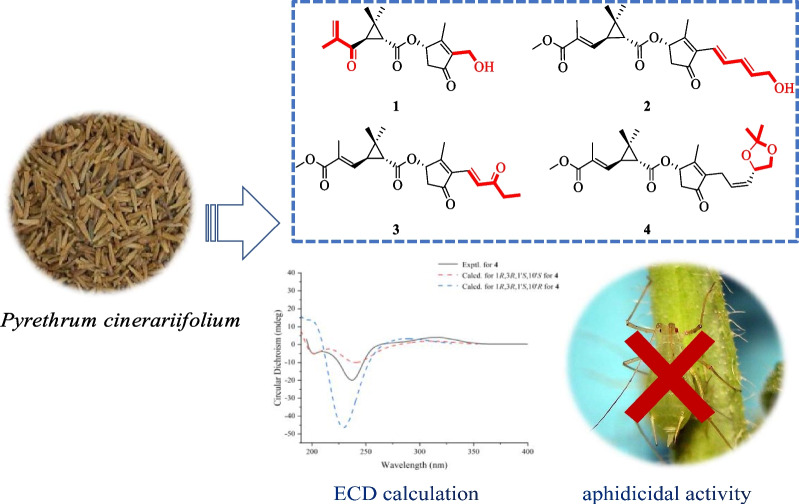

**Supplementary Information:**

The online version contains supplementary material available at 10.1007/s13659-023-00385-0.

## Introduction

In agriculture, pests can adversely affect the quality and quantity of the crops production [1]. Currently, pests control mainly relies on the use of chemical synthetic pesticides [2]. Although chemical synthesis pesticides can bring significant benefits to agricultural production in a short time, it is easy to cause environmental pollution and pesticide chemical residues of pesticides [3, 4]. Therefore, it is worthwhile to find and develop ecologically safe pesticides. Previous studies have found that many plant-derived pesticides, including pyrethrin, marine and rotenone, are low toxicity, safe, efficient and easily degradable [5–8].

**Fig. 1:**
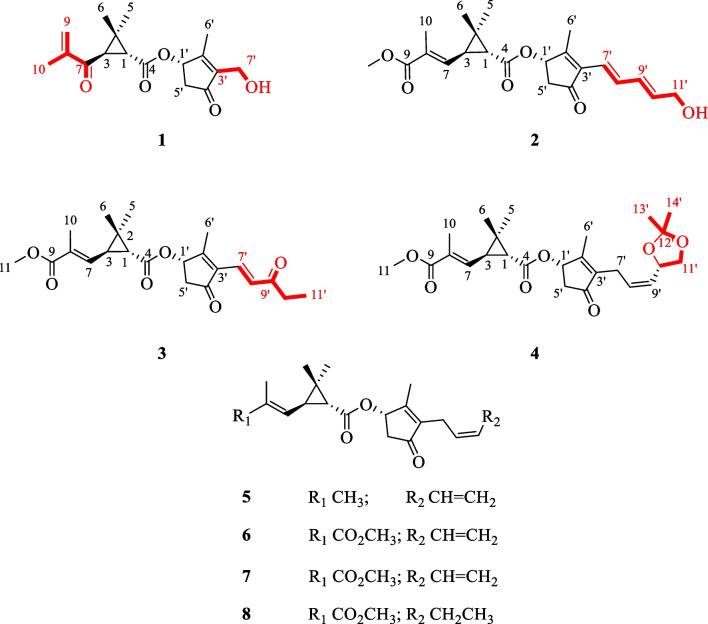
Structures of compounds **1**–**8**

Pyrethrins from *Pyrethrum* *cinerariifolium* Trev., which are mainly composed of six compounds (pyrethrin I and pyrethrin II, cinerin I and cinerin II, jasmolin I and jasmolin II) with similar structures, are representative excellent insecticidal chemicals [9, 10]. Pyrethrins have tactile toxicity to many agricultural pests, including aphids, weevils, mosquitoes and thrips, by acting on Na^+^ channels in the insect nervous system [11–14]. In addition, pyrethrins have a short half-life of about 2 h and don’t leave toxic residues in the environment, so it is commonly recognized as an environmentally friendly pesticide [15]. To find out more chemical constituents of pyrethrins with insecticidal activity, we conducted further phytochemical studies on *P.* *cinerariifolium* seeds. In this research, eight pyrethrins (Fig. 1) were identified, including four undescribed compounds and four known compounds, and their insecticidal activities were evaluated.

## Results and discussion

### Structural identification of compounds

**Fig. 2:**
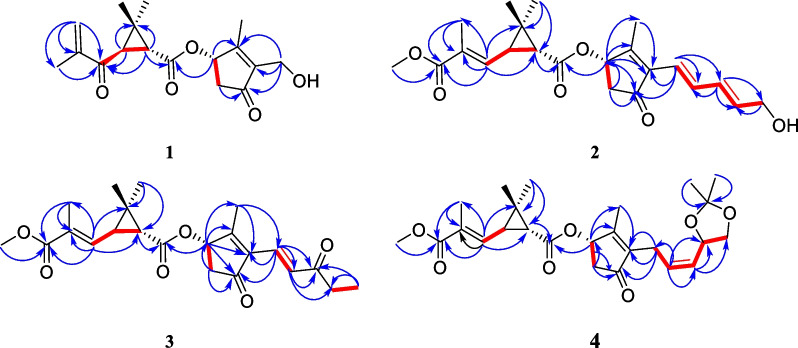
Key HMBC (blue arrows) and ^1^H-^1^H COSY (red line) correlations of **1**, **2**, **3**, and **4**

**Fig. 3:**
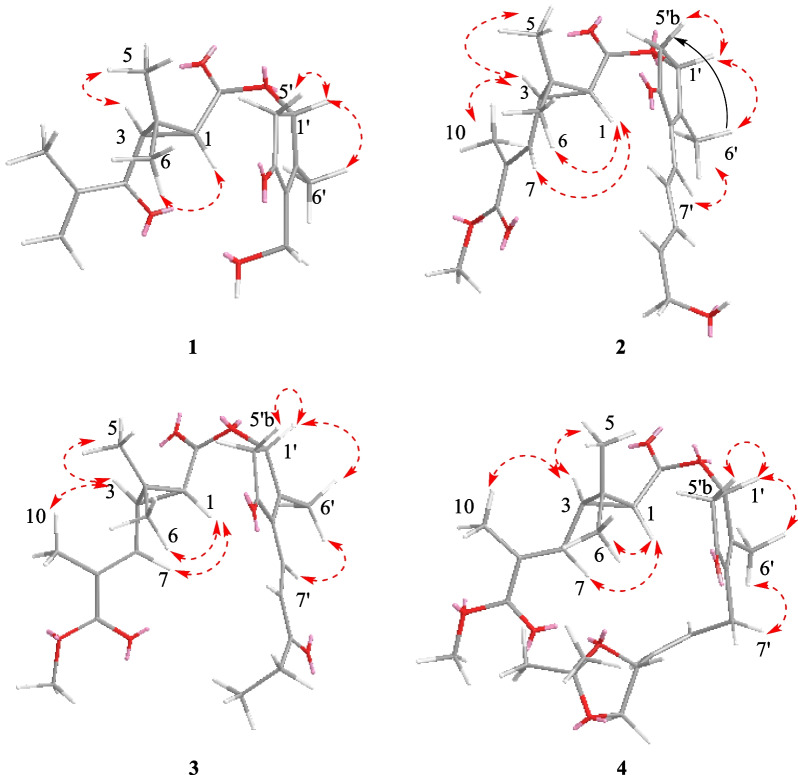
Key ROESY (red dotted arrows) correlations of **1**, **2**, **3**, and **4**

**Fig. 4:**
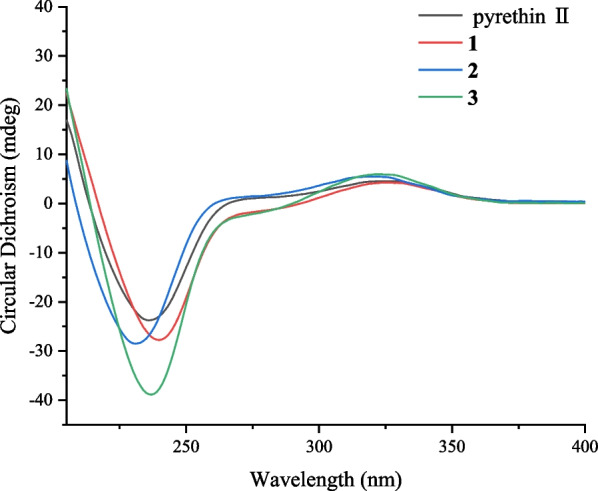
Experimental ECD spectrum of pyrethin II, **1**, **2**, and **3**

Pyrethrin C (**1**) was a colorless oil. The HRESIMS ion peak at *m/z* 305.1395 ([M‒H]^‒^, calcd for 305.1394) indicated its molecular formula as C_17_H_22_O_5_. The characteristic signals for protons were observed in ^1^H NMR spectrum (Table 1), included three methyl groups (*δ*_H_ 1.09, s; *δ*_H_ 1.36, s; *δ*_H_ 2.11, s), a methylene group (*δ*_H_ 2.26, dd, *J* = 18.8, 2.3 Hz; *δ*_H_ 2.93, dd, *J* = 18.8, 6.3 Hz), and three methine groups (*δ*_H_ 2.89, d, *J* = 5.2 Hz; *δ*_H_ 2.45, d, *J* = 5.2 Hz; *δ*_H_ 5.65, d, *J* = 6.3 Hz). Besides, the ^13^C and DEPT NMR spectra showed 17 carbon signals, attributed to carbons of four methyls, three methylenes (an olefinic, an oxygenated and an aliphatic methylenes), and three methines (an oxygenated and two aliphatic methine), as well as seven non-protonated carbons (three olefinic and three carbonyls). The aforementioned information proved that compound **1** was similar to the 4(*S*)-1-oxo-2-allyl-3-methyl-2-cyclopente-4-yl-2,2-dimethyl-3(*R*)-(2-methyl-1-propenyl)-1(*R*)-cyclopropane carboxylate [16]. The main differences between them were that the C-7 (*δ*_C_ 196.9) was a carbonyl instead of a methylene, and the vinylene attached to the C-7ʹ was replaced by the OH group in 1D NMR spectra of **1**. The above inference can be unambiguously verified by the HMBC cross-peaks (Fig. 2) of H-9a (*δ*_H_ 6.02), H-10 (*δ*_H_ 1.90), H-3 (*δ*_H_ 2.45), and H-1 (*δ*_H_ 2.89) with the carbonyl (C-7); of H-7ʹ (*δ*_H_ 4.38) with C-3ʹ (*δ*_C_ 141.5), C-4ʹ (*δ*_C_ 205.1) and C-6ʹ (*δ*_C_ 14.0). In the ROESY spectra (Fig. 3), the correlations of H-1 and H-6, H-3 and H-5 indicated the protons of H-1 was *β*-oriented and H-3 was *α*-oriented. Moreover, the ROESY correlations of H-6ʹ with H-5ʹb, H-7ʹ, H-1ʹ, and H-1ʹ with H-5ʹb indicated that these protons were co-facial and arbitrarily assigned as *β*-orientation, as well as H-5ʹa was *α*-orientation. Meanwhile, the analysis of reported pyrethrins combined with biosynthetic pathways could confirm that H-1ʹ was generally *β*-orientation [9, 17, 18]. In addition, the ROESY correlations of H-1 with H-7 and H-3 and H-10 manifested that Δ^7,8^-double bond was *E*-configuration. The above ROESY correlations and the experimental ECD spectra (Fig. 4) indicated the stereotypic configuration of (1*R*, 3*R*, and 1ʹ*S*), consistent with known pyrethrin II in previous studies [17, 19]. Therefore, the structure of **1** was determined and named pyrethrin C.

**Table 1:** ^1^H NMR data of compounds **1**–**4** (*δ* in ppm, *J* in Hz)

**Position**	**1**^a^	**2**^b^	**3**^b^	**4**^a^
1	2.89 d (5.2)	1.75 d (5.2)	1.76 d (5.2)	1.73 d (5.2)
3	2.45 d (5.2)	2.24 dd (9.6, 5.2)	2.25 dd (9.6, 5.2)	2.20 m
5	1.09 s	1.31 s	1.25 s	1.24 s
6	1.36 s	1.24 s	1.32 s	1.30 s
7		6.46 d (9.7)	6.46 d (9.7)	6.46 d (9.7)
9a	6.02 s			
9b	5.88 s			
10	1.90 s	1.95 s	1.95 s	1.95 s
11		3.74 s	3.75 s	3.73 s
1ʹ	5.65 d (6.3)	5.68 d (6.3)	5.72 d (6.3)	5.65 d (6.3)
5ʹa	2.93 dd (18.8, 6.3)	5.93 dd (18.8, 6.3)	2.98 dd (18.8, 6.3)	2.87 dd (18.8, 6.3)
5ʹb	2.26 dd (18.8, 2.3)	5.29 dd (18.8, 2.3)	2.34 dd (18.8, 2.3)	2.23 dd (18.8, 2.3)
6ʹ	2.11 s	2.11 s	2.22 s	2.05 s
7ʹa	4.38 m	6.21 d (15.7)	7.24 d (15.9)	3.02 dd (14.7, 7.3)
7ʹb				3.12 dd (14.7, 7.3)
8ʹ		7.42 dd (15.7, 10.8)	7.43 d (15.9)	5.50 dd (10.9, 7.3)
9ʹ		6.34 dd (15.3, 10.8)		5.48 m
10ʹ		6.05 dd (15.3, 5.6)	2.64 q (7.2)	5.00 dd (8.0, 6.0)
11ʹa		4.26 d (5.6)	1.14 t (7.2)	3.53 t (7.9)
11ʹb				4.14 dd (8.1, 4.0)
13ʹ				1.43 s
14ʹ				1.41 s

^a^Measured at 800 MHz

^b^Measured at 600 MHz

Pyrethrin D (**2**) was a yellow oil, and its molecular formula was identified as C_22_H_28_O_6_ through HRESIMS ion at *m/z* 387.1395 ([M‒H]^‒^, calculated. 387.1394) with nine degrees of unsaturation. Carefully analyze the 1D NMR spectroscopic data (Table 2) can be found in the similarity of **2** and pyrethrin II (**6**) [19]. Compared with pyrethrin II (**6**), the main differences of **2** were that the positions of conjugate double bonds, which transferred from C-8ʹ/C-9ʹ and C-10ʹ/C-11ʹ to C-7ʹ/C-8ʹ and C-9ʹ/C-10ʹ, and the terminal carbon C-11ʹ was attached to a hydroxyl group, which was proved by the long-range ^1^H-^1^H COSY correlations (Fig. 2) of H-7/H-8/H-9/H-10/H-11. Simultaneously, the above deduction also could be proved by the key HMBC cross-peaks (Fig. 2) from H-7ʹ (*δ*_H_ 6.21) to C-2ʹ (*δ*_C_ 164.2), C-3ʹ (*δ*_C,_ 137.6), C-4ʹ (*δ*_C_ 202.9), and C-8ʹ (*δ*_C_ 135.1); H-9ʹ (*δ*_H_ 6.34) to C-8ʹ (*δ*_C_ 135.1), C-10ʹ (*δ*_C_ 135.7), and C-11ʹ (*δ*_C_ 63.2); H-11ʹ (*δ*_H_ 4.26) to C-10ʹ (*δ*_C_ 135.7). Besides, according to the ^1^H NMR data (Table 1), the large value of the coupling constant *J*_7'-8'_ (15.7 Hz > 15.0 Hz) and *J*_9'-10'_ (15.3 Hz > 15.0 Hz) suggested their *E*-configurations. In addition, the ROESY correlations (Fig. 3) of H-1 with H-7 and H-3 with H-10 manifested that Δ^7,8^-double bond was *E*-configuration. Upon carefully analyzing the ROESY spectra correlations (H-1 with H-6, H-3 with H-5, H-6ʹ with H-5ʹb, H-7ʹ, H-1ʹ, H-1ʹ with H-5ʹb), and combined with the biosynthesis pathway and the experimental ECD spectra (Fig. 4), which indicated that **2** had the same stereoscopic configuration with pyrethin II (**6**). The above information indicated that the structure of pyrethrin D (**2**) as shown in Fig. 1.

**Table 2:** ^13^C NMR spectroscopic data of compounds **1**–**4** (*δ* in ppm, CDCl_3_)

**Position**	**1**^a^	**2**^b^	**3**^b^	**4**^a^
1	38.4, CH	35.7, CH	35.8, CH	35.7, CH
2	32.6, C	30.6, C	31.0, C	30.6, C
3	32.2, CH	33.0, CH	33.4, CH	32.9, CH
4	170.7, C	171.1, C	171.3, C	171.2, C
5	19.9, CH_3_	20.3, CH_3_	22.6, CH_3_	22.3, CH_3_
6	20.1, CH_3_	21.9, CH_3_	20.7, CH_3_	20.4, CH_3_
7	196.9, C	138.9, CH	138.9, CH	138.9, CH
8	145.5, C	129.9, C	130.1, C	129.8, C
9	126.2, CH_2_	168.1, C	168.3, C	168.1, C
10	17.3, CH_3_	13.0, CH_3_	13.1, CH_3_	12.9, CH_3_
11		51.9, CH_3_	52.1, CH_3_	51.8, CH_3_
1ʹ	73.4, CH	73.0, CH	72.9, CH	73.4, CH
2ʹ	166.6, C	164.2, C	172.2, C	164.9, C
3ʹ	141.5, C	137.6, C	136.1, C	141.7, C
4ʹ	205.1, C	202.9, C	202.3, C	203.4, C
5ʹ	42.0, CH_2_	42.7, CH_2_	42.8, CH_2_	41.9, CH_2_
6ʹ	14.0, CH_3_	14.3, CH_3_	14.8, CH_3_	14.1, CH_3_
7ʹ	55.1, CH_2_	120.2, CH	128.1, CH	21.9, CH_2_
8ʹ		135.1, CH	130.8, CH	129.2, CH
9ʹ		131.6, CH	201.6, C	129.0, CH
10ʹ		135.7, CH	35.9, CH_2_	71.6 CH
11ʹ		63.2, CH_2_	8.1, CH_3_	69.4, CH_2_
12ʹ				109.2, C
13ʹ				25.9, CH_3_
14ʹ				26.8, CH_3_

^a^Measured at 200 MHz

^b^Measured at 150 MHz

Pyrethrin E (**3**) was obtained as a yellow oil and its molecular formula was identified as C_22_H_28_O_6_ (nine degrees of unsaturation) in terms of HRESIMS data at *m/z* 387.1817 ([M‒H]^‒^, calcd for 387.1813). Comparing the ^1^H and ^13^C NMR data, it could be found that the structure of compound **3** was similar to the isopyrethrin II [20], except the absence of one double-bonded between C-9ʹ and C-10ʹ in **3**, which was replaced by the carbonyl (*δ*_C_ 201.6) located at C-9ʹ. Supporting evidence was discovered in the 2D NMR, the HMBC correlations (Fig. 2) of C-9ʹ (*δ*_C_ 201.6) with H-7ʹ (*δ*_H_ 7.24), H-8ʹ (*δ*_H_ 7.43), H-10ʹ (*δ*_H_ 2.64), H-11ʹ (*δ*_H_ 1.14), and C-8ʹ (*δ*_C_ 130.8), of C-10ʹ (*δ*_C_ 35.9) with H-8ʹ (*δ*_H_ 7.43) and H-11ʹ (*δ*_H_ 1.14), combined with the ^1^H-^1^H COSY correlation (Fig. 2) of H-10ʹ and H-11ʹ, confirmed the aforementioned deduction. The ROESY correlations (Fig. 3) of H-1 with H-7 and H-3 with H-10 manifested that Δ^7,8^-double bond was *E*-configuration. In addition, the ROESY correlations (of H-1 with H-6, H-3 with H-5, H-6ʹ with H-5ʹb, H-7ʹ, H-1ʹ, H-1ʹ with H-5ʹb) and the same ECD spectra (Fig. 4) indicated **3** had the same stereoscopic configuration with pyrethin II (**6**). Thus, the structure of **3** was established and named pyrethrin E.

At the same time, the experimental ECD spectra (Fig. 4) further proved that compounds **1**, **2**, **3** and pyrethin II (**6**) had the same Cotton effect, which can be determined that their stereoscopic configuration was consistent.

**Fig. 5:**
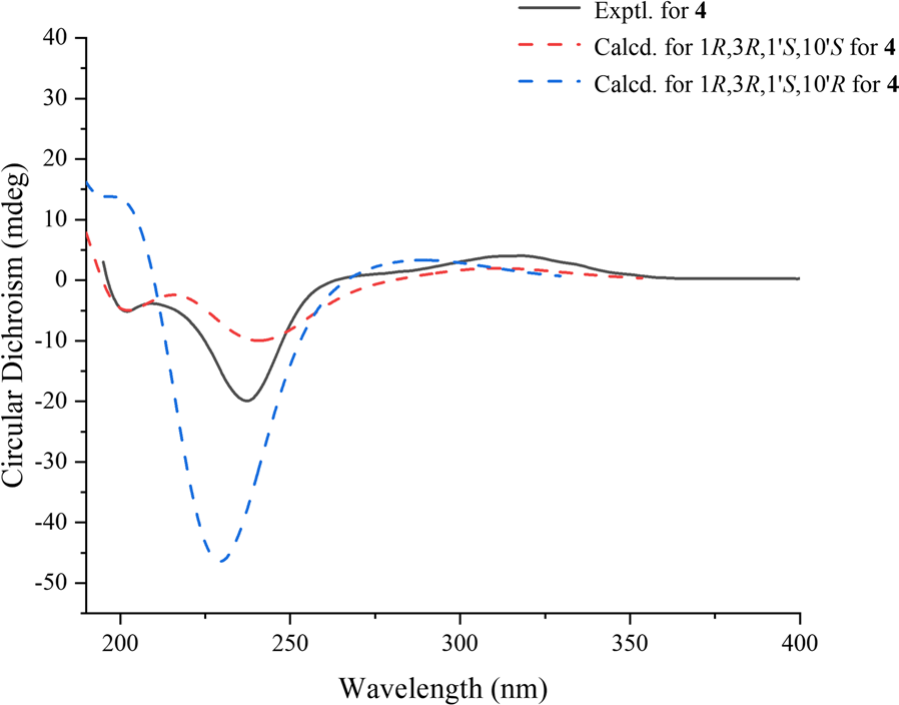
Experimental ECD spectrum of **4** and calculated ECD spectrum of (1*R*,3*R*,1′*S*,10′*S*)-**4** and (1*R*,3*R*,1′*S*,10′*R*)-**4**

Compound **4** was isolated as a yellow oil. It had a molecular formula of C_25_H_34_O_7_ based on ion peak at *m/z* 469.2197 ([M + Na]^+^, calcd for 469.2201) as given by HRESIMS data. Meanwhile, there were nine degrees of unsaturation in **4**. A detailed comparison of the ^1^H and ^13^C NMR spectroscopic data (Tables 1, 2) of **4** with the 10ʹ,11ʹ-dihydroxypyrethrin II [18] revealed that there were three additional carbon signals including two methyl signals (*δ*_C_ 25.9, C-13ʹ; *δ*_C_ 26.8, C-14ʹ) and a quaternary carbon signal (*δ*_C_ 109.2, C-12ʹ) indicated an *O*-isopropyl motif was presented in **4**. The HMBC correlations (Fig. 2) from H-13ʹ (*δ*_H_ 1.43), H-14ʹ (*δ*_H_ 1.41) to C-12ʹ (*δ*_C_ 109.2) determined the position of *O*-isopropyl motif. The planar structure of **4** was thereby finally established. The ROESY correlations (Fig. 3) of H-1 with H-7, H-3 with H-10 manifested that Δ^7,8^-double bond was *E*-configuration. In addition, the correlations of H-1 with H-6, H-3 with H-5, H-6ʹ with H-5ʹb, H-7ʹ, H-1ʹ, H-1ʹ with H-5ʹb in ROESY spectrum indicated that the relative configuration of H-1, H-3 and H-1ʹ were consistent with **1**. Thus, H-1 and H-1ʹ were identified as *β*-orientation and H-3 was *α*-orientation. However, the stereoscopic configuration of H-10ʹ could not be determined by the ROESY correlation, the ECD calculations were performed to determine it. Finally, the consistency between the calculated ECD result of (1*R*, 3*R*, 1ʹ*S*, and 10ʹ*S*) and the experimental ECD (Fig. 5) determined the absolute stereochemistry of pyrethin F (**4**).

The identification of other known compounds, including pyrethin I (**5**), pyrethin II (**6**), cinerin II (**7**) and jasmolin II (**8**), were determined by comparison their 1D NMR data with the reported compounds [19].

### Insecticidal activity

The aphidicidal activities of compounds **1**–**4** were evaluated at a concentration of 0.1 mg/mL. The results (Table 3) showed that the 24 h mortality rate of compounds **1**, **2**, **3**, and **4** ranged from 17.64% to 52.94%, which were slightly lower than the mortality rate of the positive control (pyrethin II, 83.52%). **2** and **4** showed the moderate activity (52.94% and 41.17%), indicating that they could be used as one of the active components of naturally obtained insecticides.

**Table3:** The aphidicidal activityof compounds **1**, **2**, **3**, and **4**

Compound	Concentration (mg/mL)	Mortality rate^a^ (%)	
		24 h	48 h
Pyrethrin II	0.1	83.52 ± 0.37	85.88 ± 0.31
**1**	0.1	32.94 ± 0.71	36.47 ± 0.68
**2**	0.1	52.94 ± 0.76	56.49 ± 0.53
**3**	0.1	17.64 ± 0.25	21.18 ± 0.61
**4**	0.1	41.17 ± 0.92	43.55 ± 1.50

^a^Data expressed as mean ± SD (n = 3)

Based on the above activity data, the structure–activity relationships were preliminarily discussed. Compared with the positive control (pyrethrin II), the activities of the compounds **1**–**4** decreased when the side chain structure of the three-membered ring or five-membered ring changed. Compounds with conjugated double bonds in C-3ʹ side chains of five-membered rings, such as **2** and pyrethrin II, showed better insecticidal activity than **1**, **3** and **4**. According to the higher activity of **2** compared with **3**, carboxyl substitution on C-9ʹ might decrease the aphidicidal activity.

## Experimental section

### General experimental procedure

Fractions were examined by TLC on silica gel GF254 plates (200–250, Qingdao Marine Chemical, Inc.), and the 10% H_2_SO_4_ in ethanol as developer. The silica gel (200–300 mesh, Qingdao Marine Chemical, Inc., Qingdao, China), reversed-phase C18 silica gel (40–60 μm, Merck, Darmstadt, Germany), and Sephadex LH-20 (Pharmacia, Stockholm, Sweden) were used as the material for column chromatography (CC) analysis. The Agilent 1100 or 1260 liquid chromatography system equipped with Agilent ZORBAX SB-C18 columns (5 μm, 4.6 × 250 mm) was used for HPLC analysis. 1D and 2D NMR spectra were obtained using the Bruker AV-600 and AV-800 spectrometers (Bruker, Zürich, Switzerland) with tetramethylsilane (TMS) as an internal standard. The Agilent UPLC system spectrometer (Agilent Technologies, Foster City, CA, USA) was used to obtain HRESIMS data. The Rudolph Autopol VI polarimeter (Hackettstown, NJ, USA) was used to obtain optical rotations. UV spectra were detected on an UV-2401 PC spectrometer (Shimadzu Corp., Japan). *Acyrthosiphon* *pisum* was obtained from Henan Quanying Insect Biology Co., LTD (Henan, China).

### Plant materials

The seeds of *Pyrethrum* *cinerariifolium* Trev. were collected in Xinjiang Province, People’s Republic of China in September 2020. The plant was authenticated by Mr. Zhong-Rong Li Senior Engineer, Kunming Institute of Botany, Chinese Academy of Sciences. The specimen (KUN. No. Q20200915) was deposited in the State Key Laboratory of Photochemistry and Plant Resources in West China, Kunming Institute of Botany, Chinese Academy of Sciences.

### Extraction and isolation

The dried *P.* *cinerariifolium* seeds (11.5 kg) were extracted with 90% acetone (25 L × 3) at room temperature. The residue (1.1 kg) which was obtained after the acetone solvent was removed by a rotary evaporator, was mixed with appropriate amount of H_2_O, and then extracted with petroleum ether (PE, 10 L × 3) and ethyl acetate (EtOAc, 10 L × 3). Then, the EtOAc part (314.9 g) was separated by silica gel CC eluted with PE/EtOAc (20:1, 10:1, 1:1, 1:10, 1:20, 1:40, and 1:50) to obtain seven fractions (Fr.I–Fr.VII).

Fr.II (5 g) was separated by silica gel CC (PE/EtOAc, 15:1, 12:1, 10:1, 8:1, 5:1) to give five fractions (Fr.II-1–Fr.II-5). Fr.II-2 (56 mg) was further purified by the semipreparative HPLC with same gradient elution to give **5** (84% MeCN/H_2_O, 6.4 mg, t_R_ = 17.6 min) and **8** (57% MeCN/H_2_O, 7.1 mg, t_R_ = 44.2 min).

The separation of Fr. III (39 g) was firstly carried out using an RP-18 column eluted with the MeOH/H_2_O (50:50, 60:40, 70:30, and 75:25), and produced six fractions (Fr. III-1–Fr. III-6). Then, Fr.III-2 (5 g) was further separated by Sephadex LH-20 column to get three fractions (Fr. III-2-1–Fr. III-2-3), in which MeOH was used as eluent. After that, all subfractions were further separated by a silica gel CC with CH_2_Cl_2_-EtOAc (15:1, 12:1, 10:1, 7:1, 5:1, 3:1, and 1:1) and purified by semipreparative HPLC eluted with CH_3_OH/H_2_O or MeCN/H_2_O to afford compounds **1** (48% CH_3_OH/H_2_O, 1.9 mg, t_R_ = 30.1 min), **6** (65% MeCN/H_2_O, 34.0 mg, t_R_ = 27.5 min), and **7** (85% MeCN/H_2_O, 5.2 mg, t_R_ = 21.2 min).

Fr. IV (29.0 g) was treated into five sub-fractions (Fr.IV-1–Fr.IV-5) using RP-18 column eluting sequentially with the solvents system of MeOH/H_2_O (30:70 to 70:30). Then, Fr.IV-1 (2 g) was fractionated into four subfractions (Fr.IV-1-1–Fr.IV-1-4) using Sephadex LH-20 column with MeOH. Fr.IV-1-3 (52 mg) was purified by semipreparative HPLC eluted with MeCN/H_2_O to give compound **3** (46% MeCN/H_2_O, 10.5 mg, t_R_ = 34.1 min). Fr.IV-3 (10 g) was further separated by silica gel CC (CH_2_Cl_2_-EtOAc, 10:1, 7:1, 5:1, 3:1, 1:1, 1:3, and 1:5) to give 12 subfractions (Fr.IV-3-1–Fr.IV-3-12). Fr.IV-3-5 (112 mg) was purified by semipreparative HPLC eluted with CH_3_CN/H_2_O to give compound **2** (32% MeCN/H_2_O, 1.5 mg, t_R_ = 36.8 min) and **4** (52% MeCN/H_2_O, 2.3 mg, t_R_ = 28.9 min).

### Compound characterization

***PyrethrinC*** **(1)**: colorless oil; C_17_H_22_O_5_; [*α*]_D_^21^ ‒14.6 (*c* 0.11, CH_3_OH); UV (MeOH) λ_max_ (logε): 309 (2.82) and 222 (3.91) nm; HRESIMS *m/z* 305.1395 [M–H]^‒^ (calcd for. 305.1394, C_17_H_21_O_5_).

***Pyrethrin*** ***D*** **(2)**: light yellow oil; C_22_H_28_O_6_; [*α*]_D_^21^ ‒20.0 (*c* 0.12, CH_3_OH); UV (MeOH) λ_max_ (logε): 238 (4.04) and 196 (3.84) nm; HRESIMS *m/z* 387.1395 [M–H]^‒^ (calcd for. 387.1394, C_22_H_28_O_6_).

***Pyrethrin*** ***E*** **(3)**: light yellow oil; C_22_H_28_O_6_; [*α*]_D_^21^ ‒33.64 (*c* 0.11, CH_3_OH); UV (MeOH) λ_max_ (logε): 257 (4.30) and 197 (4.11) nm; HRESIMS *m/z* 387.1817 [M–H]^‒^ (calcd for. 387.1813, C_22_H_28_O_6_).

***Pyrethrin*** ***F*** **(4)**: light yellow oil; C_25_H_34_O_7_; [*α*]_D_^21^ ‒28.4 (*c* 0.10, CH_3_OH); UV (MeOH) λ_max_ (logε): 233 (4.24) and 197 (3.97) nm; HRESIMS *m/z* 469.2197 [M + Na]^+^ (calcd for. 469.2201, C_25_H_34_O_7_).

The ^1^H NMR data were shown in Table 1, ^13^C NMR data were shown in Table 2, and 2D NMR data (HSQC, HMBC, ^1^H-^1^H COSY, ROESY) were shown in Additional file 1 for pyrethrin (C–F).

### Bioassay

The contact toxicity assay was carried out according to the previous method with some modifications [21]. The same-size *A.pisum* adults were collected into the insect culture box with fresh pea seedlings and moist tissue paper for experimentation. The insect culture box was sealed with plastic wrap and left holes for air permeability. The test samples were accurately measured and dissolved in acetone to prepare sample solutions of 0.1 mg/mL. Then 2 μL of the diluted acetone solutions were applied to the dorsal thorax of the *A.pisum* adults. 30 *A.pisum* adults were used for each group, and each experiment was replicated three times. The *A.pisum* adults of each treatment were transferred to the corresponding insect culture box. The mortality was evaluated after 24 h and 48 h. If the *A.pisum* could not move when disturbed by a wet brush, they were considered dead. Pyrethrin II was used as a positive control, and acetone treatments were determined as a blank control. Mortality was corrected by Abbott's formula [22].

### ECD calculations

The experimental ECD spectra of the compounds were recorded in MeOH. The ECD calculation was performed as previously reported [23]. The specific calculation process and data were described in the Additional file 1.

## Conclusion

In summary, eight pyrethrins were isolated from seeds of *P.* *cinerariifolium*, including four new compounds (**1**–**4**) and four known compounds (**5**–**8**). All of them had the same core structure as pyrethrins, in which the side chain on C-3 and C-3ʹ of pyrethrin C (**1**) was changed, and the side chain on C-3ʹ of pyrethrin D (**2**), pyrethrin E(**3**), and pyrethrin F (**4**) were mainly changed. Meanwhile, we also tested the aphidicidal activities of **1**, **2**, **3**, and **4**, and the results exhibited that they all had aphidicidal activity, among which pyrethrin D (**2**) had the strongest mortality rate of 52.94%. These findings suggested that the *P.* *cinerariifolium* can be used as an important source of the insecticidal ingredient and continue to develop insecticides for agricultural production.

## Supplementary Information

**Additional** **file** **1:** **Figure** **S1**. ^1^H NMR spectrum (800 MHz) of compound **1** in CD_3_Cl. **Figure** **S2**. ^13^C NMR spectrum (200 MHz) of compound **1** in CD_3_Cl. **Figure** **S3.** HSQC spectrum of compound **1** in CD_3_Cl. **Figure** **S4.** HMBC spectrum of compound **1** in CD_3_Cl. **Figure** **S5.** ^1^H-^1^H COSY spectrum of compound **1** in CD_3_Cl. **Figure** **S6.** ROESY spectrum of compound **1** in CD_3_Cl. **Figure** **S7.** HRESI (-) MS spectrum of compound **1.** **Figure** **S8.** OR of compound **1.** **Figure** **S9.** UV spectrum of compound **1.** **Figure** **S10.** ^1^H NMR spectrum (600 MHz) of compound **2** in CD_3_Cl. **Figure** **S11.** ^13^C NMR spectrum (150 MHz) of compound **2** in CD_3_Cl. **Figure** **S12.** HSQC spectrum of compound **2** in CD_3_Cl. **Figure** **S13.** HMBC spectrum of compound **2** in CD_3_Cl. **Figure** **S14.** ^1^H-^1^H COSY spectrum of compound **2** in CD_3_Cl. **Figure** **S15.** ROESY spectrum of compound **2** in CD_3_Cl. **Figure** **S16.** HRESI (-) MS spectrum of compound **2.** **Figure** **S17.** OR of compound **2.** **Figure** **S18.** UV spectrum of compound **2.** **Figure** **S19.** ^1^H NMR spectrum (600 MHz) of compound **3** in CD_3_Cl. **Figure** **S20.** ^13^C NMR spectrum (150 MHz) of compound **3** in CD_3_Cl. **Figure** **S21.** HSQC spectrum of compound **3** in CD_3_Cl. **Figure** **S22.** HMBC spectrum of compound **3** in CD_3_Cl. **Figure** **S23.** ^1^H-^1^H COSY spectrum of compound **3** in CD_3_Cl. **Figure** **S24.** ROESY spectrum of compound **3** in CD_3_Cl. **Figure** **S25.** HRESI (-) MS spectrum of compound **3.** **Figure** **S26.** OR of compound **3.** **Figure** **S27.** UV spectrum of compound **3.** **Figure** **S28.** ^1^H NMR spectrum (800 MHz) of compound **4** in CD_3_Cl. **Figure** **S29.** ^13^C NMR spectrum (200 MHz) of compound **4** in CD_3_Cl. **Figure** **S30.** HSQC spectrum of compound **4** in CD_3_Cl**.** **Figure** **S31.** HMBC spectrum of compound **4** in CD_3_Cl. **Figure** **S32.** ^1^H-^1^H COSY spectrum of compound **4** in CD_3_Cl. **Figure** **S33.** ROESY spectrum of compound **4** in CD_3_Cl. **Figure** **S34.** HRESI (+) MS spectrum of compound **4.** **Figure** **S35.** OR of compound **4**. **Figure** **S36.** UV spectrum of compound **4.** **Figure** **S37.** Five optimized conformers of **4-1.** **Table** **S1**. Conformational analysis of the eight optimized conformers of **4-1** in the gas phase (T = 298.15 K). **Table** **S2**. Atomic coordinates (Å) of **4-1a** obtained at the CAM-B3LYP/TZVP level of theory in the MeOH. **Table** **S3.** Atomic coordinates (Å) of **4-1b** obtained at the CAM-B3LYP/TZVP level of theory in the MeOH. **Table** **S4.** Atomic coordinates (Å) of **4-1c** obtained at the CAM-B3LYP/TZVP level of theory in the MeOH. **Table** **S5.** Atomic coordinates (Å) of **4-1d** obtained at the CAM-B3LYP/TZVP level of theory in the MeOH. **Table** **S6.** Atomic coordinates (Å) of **4-1e** obtained at the CAM-B3LYP/TZVP level of theory in the MeOH. **Figure** **S38.** Seven optimized conformers of **4-2.** **Table** **S7.** Conformational analysis of the eight optimized conformers of **4-2** in the gas phase (T = 298.15 K). **Table** **S8.** Atomic coordinates (Å) of **4-2a** obtained at the CAM-B3LYP/TZVP level of theory in the MeOH. **Table** **S9.** Atomic coordinates (Å) of **4-2b** obtained at the CAM-B3LYP/TZVP level of theory in the MeOH. **Table** **S10.** Atomic coordinates (Å) of **4-2c** obtained at the CAM-B3LYP/TZVP level of theory in the MeOH. **Table** **S11.** Atomic coordinates (Å) of **4-2d** obtained at the CAM-B3LYP/TZVP level of theory in the MeOH. **Table** **S12.** Atomic coordinates (Å) of **4-2e** obtained at the CAM-B3LYP/TZVP level of theory in the MeOH. **Table** **S13.** Atomic coordinates (Å) of **4-2f** obtained at the CAM-B3LYP/TZVP level of theory in the MeOH. **Table** **S14.** Atomic coordinates (Å) of **4-2g** obtained at the CAM-B3LYP/TZVP level of theory in the MeOH.

## Data Availability

The data that support the findings of this study were available on request from the corresponding author, upon reasonable request.
