# Supplementary material for: Four undescribed pyrethrins from seeds of Pyrethrum cinerariifolium and their aphidicidal activity
Source: Nat Prod Bioprospect. 2023 Jul 7;13(1):22. doi: 10.1007/s13659-023-00385-0 (PMC10326172; doi:10.1007/s13659-023-00385-0)
Supplement: Supplementary file 1 — Additional file 1: Figure S1. 1H NMR spectrum (800 MHz) of compound 1 in CD3Cl. Figure S2. 13C NMR spectrum (200 MHz) of compound 1 in CD3Cl. Figure S3. HSQC spectrum of compound 1 in CD3Cl. Figure S4. HMBC spectrum of compound 1 in CD3Cl. Figure S5. 1H-1H COSY spectrum of compound 1 in CD3Cl. Figure S6. ROESY spectrum of compound 1 in CD3Cl. Figure S7. HRESI (-) MS spectrum of compound 1. Figure S8. OR of compound 1. Figure S9. UV spectrum of compound 1. Figure S10. 1H NMR spectrum (600 MHz) of compound 2 in CD3Cl. Figure S11. 13C NMR spectrum (150 MHz) of compound 2 in CD3Cl. Figure S12. HSQC spectrum of compound 2 in CD3Cl. Figure S13. HMBC spectrum of compound 2 in CD3Cl. Figure S14. 1H-1H COSY spectrum of compound 2 in CD3Cl. Figure S15. ROESY spectrum of compound 2 in CD3Cl. Figure S16. HRESI (-) MS spectrum of compound 2. Figure S17. OR of compound 2. Figure S18. UV spectrum of compound 2. Figure S19. 1H NMR spectrum (600 MHz) of compound 3 in CD3Cl. Figure S20. 13C NMR spectrum (150 MHz) of compound 3 in CD3Cl. Figure S21. HSQC spectrum of compound 3 in CD3Cl. Figure S22. HMBC spectrum of compound 3 in CD3Cl. Figure S23. 1H-1H COSY spectrum of compound 3 in CD3Cl. Figure S24. ROESY spectrum of compound 3 in CD3Cl. Figure S25. HRESI (-) MS spectrum of compound 3. Figure S26. OR of compound 3. Figure S27. UV spectrum of compound 3. Figure S28. 1H NMR spectrum (800 MHz) of compound 4 in CD3Cl. Figure S29. 13C NMR spectrum (200 MHz) of compound 4 in CD3Cl. Figure S30. HSQC spectrum of compound 4 in CD3Cl. Figure S31. HMBC spectrum of compound 4 in CD3Cl. Figure S32. 1H-1H COSY spectrum of compound 4 in CD3Cl. Figure S33. ROESY spectrum of compound 4 in CD3Cl. Figure S34. HRESI (+) MS spectrum of compound 4. Figure S35. OR of compound 4. Figure S36. UV spectrum of compound 4. Figure S37. Five optimized conformers of 4-1. Table S1. Conformational analysis of the eight optimized conformers of 4-1 in the gas phase (T = 298.15 K). Table S2. Atomic coordinates (Å) of [file 13659_2023_385_MOESM1_ESM.docx]

**Supplementary Material for**

**Four undescribed pyrethrins from seeds of *Pyrethrum cinerariifolium* and their aphidicidal activity**

Hao-Ran Zhou^a,b^, Li-Wu Lin^a,b^, Zhong-Rong Li^a^, Xing-Rong Peng^a,b^, Ming-Hua Qiu* ^a,b^

^a^ State Key Laboratory of Phytochemistry and Plant Resources in West China, Kunming Institute of Botany, Chinese Academy of Sciences, Kunming 650201, PR China

^b^ University of Chinese Academy of Sciences, Beijing 100049, PR China

* Corresponding author.

Telephone: +86-0871-65223327. Fax: +86-0871-65223325. E-mail: mhchiu@mail.kib.ac.cn

**Contents**

| 1. 1D, 2D NMR, HRESIMS, OR and UV spectra of **1** | 3-10 |
| --- | --- |
| 1. 1D, 2D NMR, HRESIMS, OR and UV spectra of **2** | 11-18 |
| 1. 1D, 2D NMR, HRESIMS, OR and UV spectra of **3** | 19-26 |
| 1. 1D, 2D NMR, HRESIMS, OR and UV spectra of **4** | 27-34 |
| 1. ECD data of compound **4** | 35-48 |

1′

6′

10

6

9b

9a

5′a

5′b

7

5

3

1

**Figure S1**. ^1^H NMR spectrum (800 MHz) of compound **1** in CD_3_Cl

2

10

5

6

6′

3

1′’

5′

7′

1′

9

3′

8

2’

4′

7

4

**Figure S2**. ^13^C NMR spectrum (200 MHz) of compound **1** in CD_3_Cl

**Figure S3.** HSQC spectrum of compound **1** in CD_3_Cl

**Figure S4.** HMBC spectrum of compound **1** in CD_3_Cl

**Figure S5.** ^1^H-^1^H COSY spectrum of compound **1** in CD_3_Cl

**Figure S6.** ROESY spectrum of compound **1** in CD_3_Cl


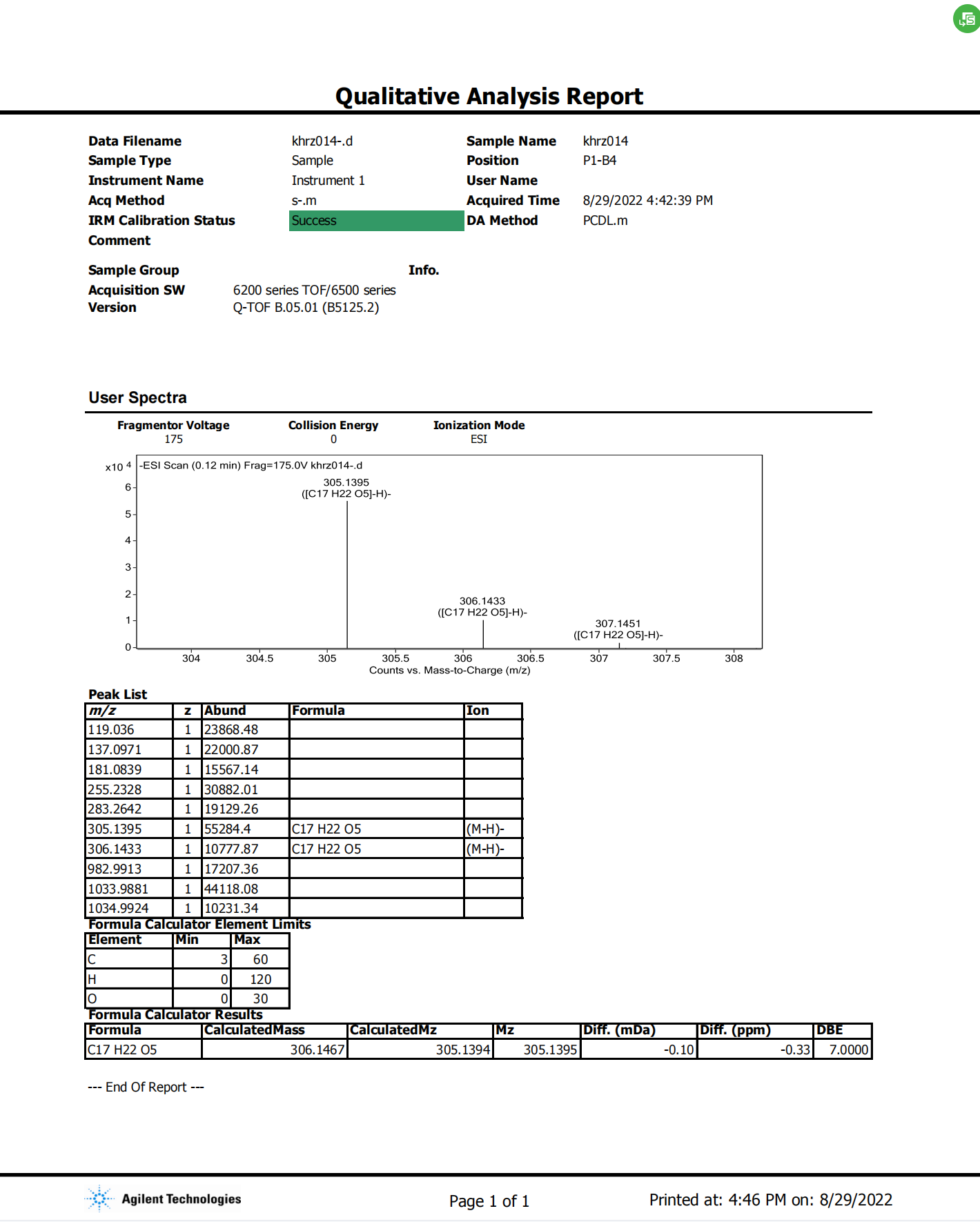


**Figure S7.** HRESI (-) MS spectrum of compound **1**

**
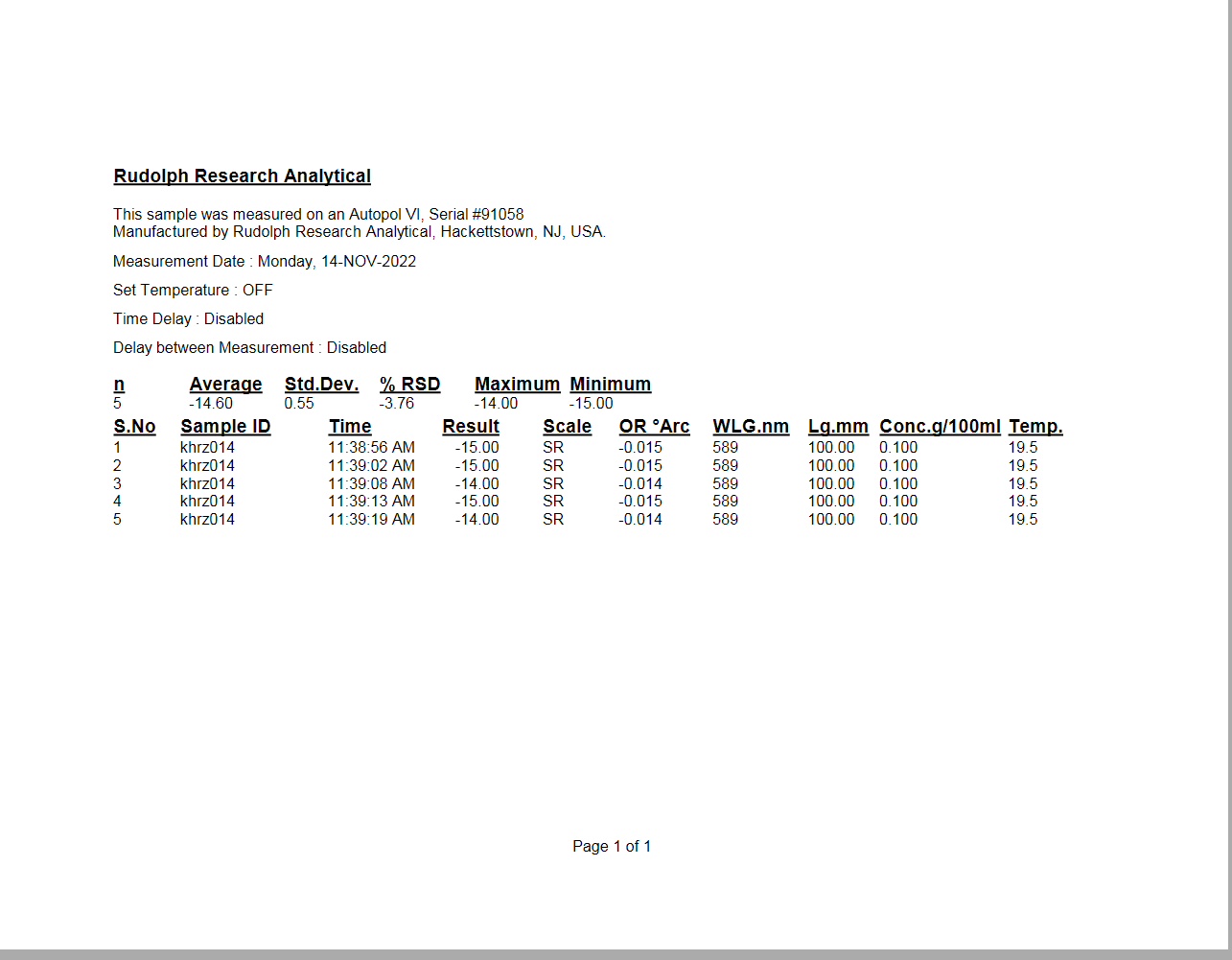
**

**Figure S8.** OR of compound **1**


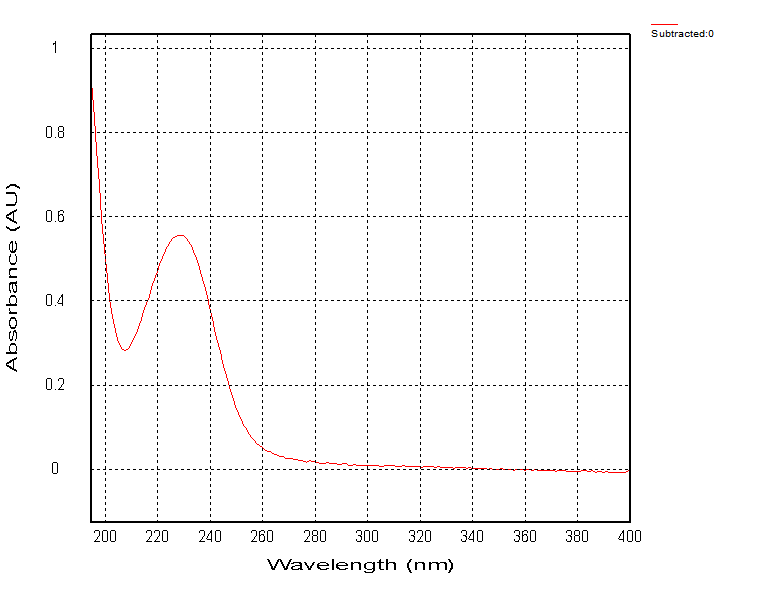


**Figure S9.** UV spectrum of compound **1**

6

5

1

10

6′

3

5′a

5′b

11’

1′

10′

7′

9′

7

8′

**Figure S10.** ^1^H NMR spectrum (600 MHz) of compound **2** in CD_3_Cl

6

9′

7′

10

6′

5

2

3

1

5′

11

1′

11′

8

3′

10′

8′

7

9

2′

4

4′

**Figure S11.** ^13^C NMR spectrum (150 MHz) of compound **2** in CD_3_Cl

**Figure S12.** HSQC spectrum of compound **2** in CD_3_Cl

**Figure S13.** HMBC spectrum of compound **2** in CD_3_Cl

**Figure S14.** ^1^H-^1^H COSY spectrum of compound **2** in CD_3_Cl

**Figure S15.** ROESY spectrum of compound **2** in CD_3_Cl


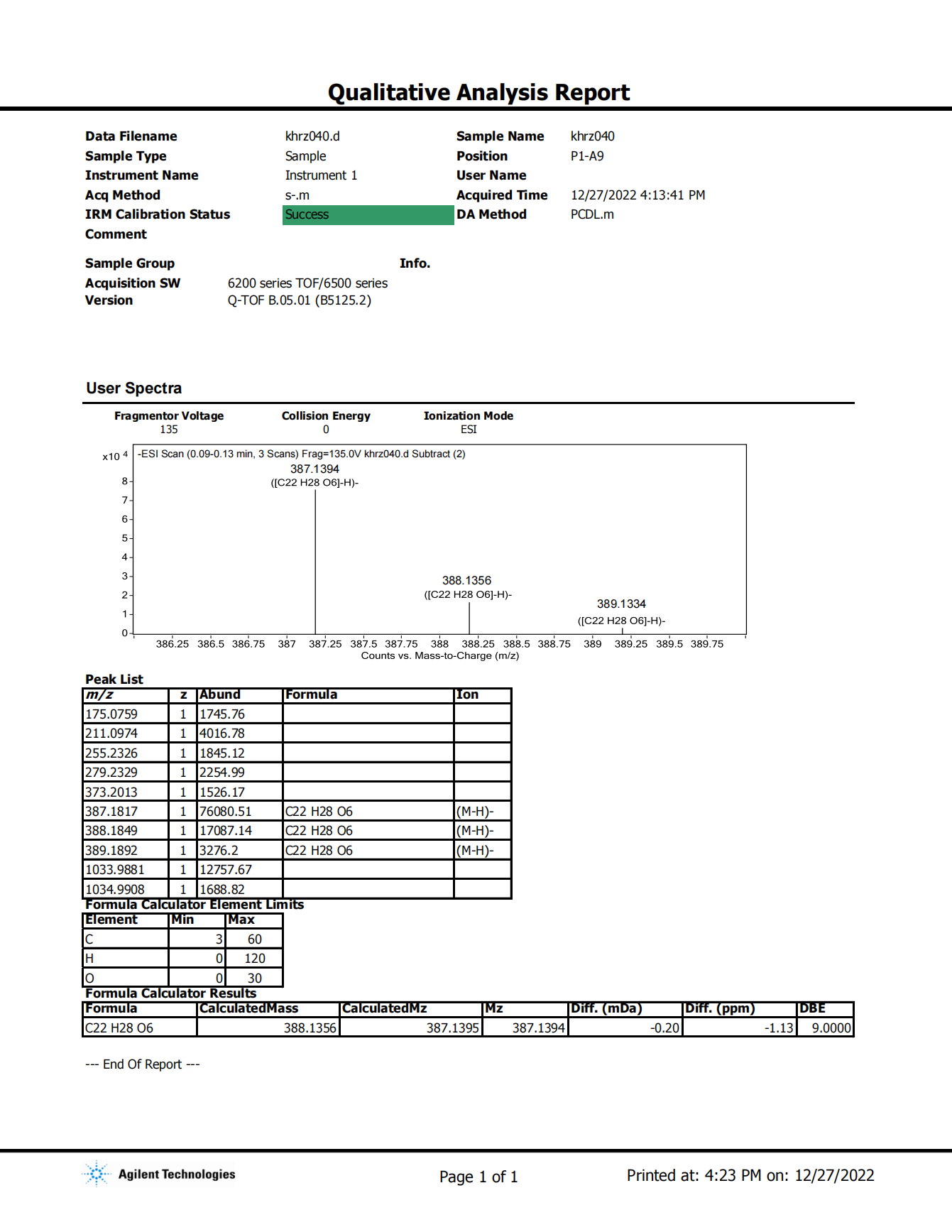


**Figure S16.** HRESI (-) MS spectrum of compound **2**


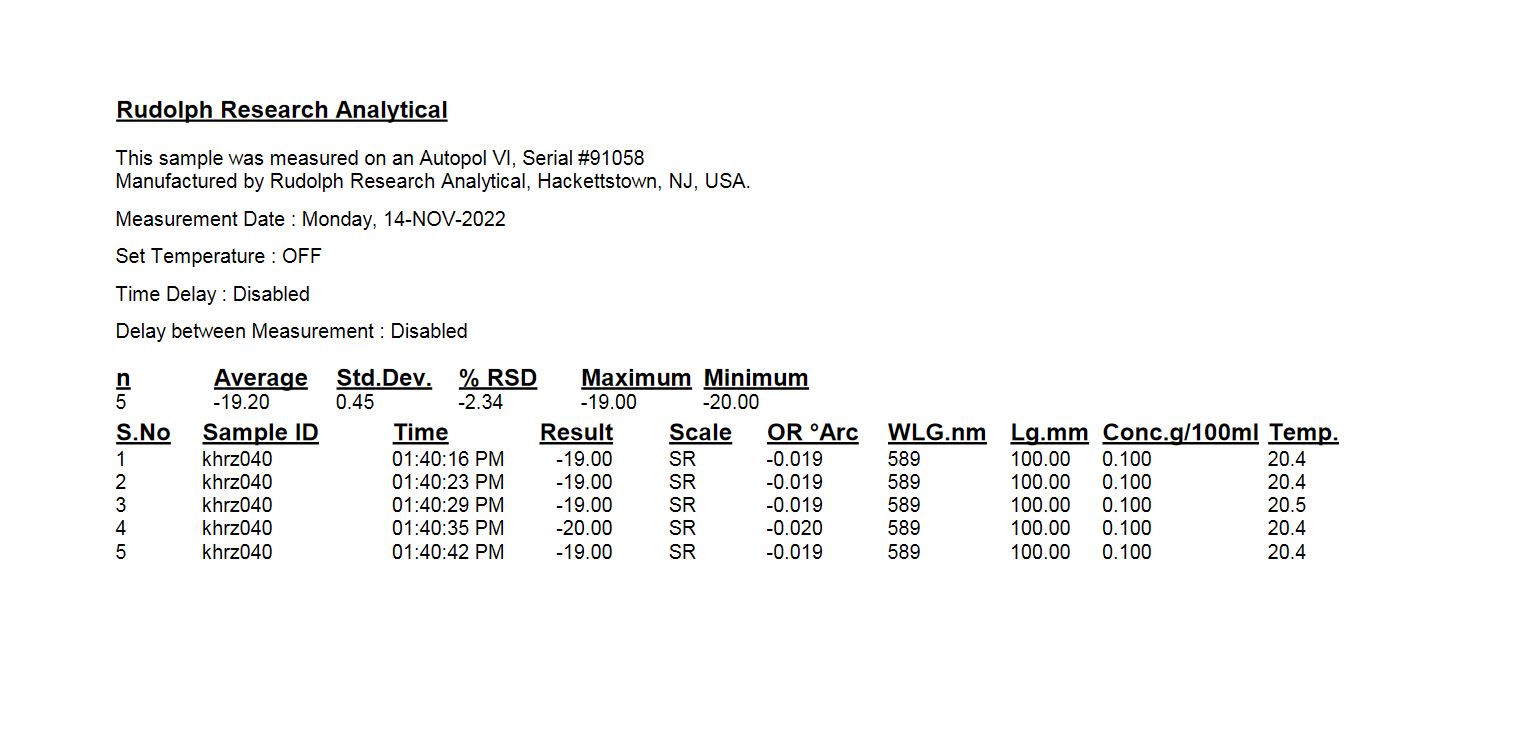


**Figure S17.** OR of compound **2**


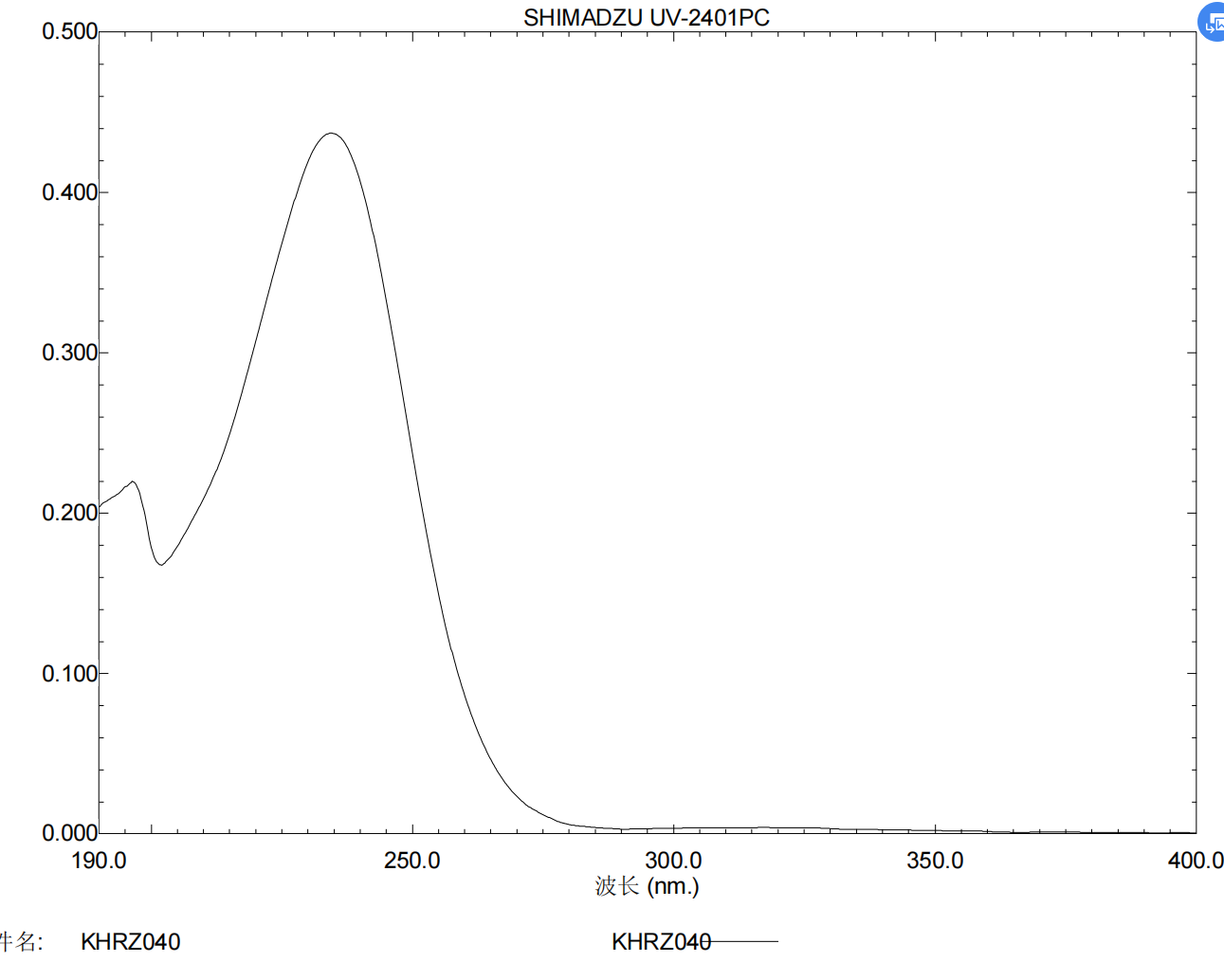


**Figure S18.** UV spectrum of compound **2**

1′

1

11

6

5

10

6′

3

5′a

10′

5′b

11

7

7′

8′

**Figure S19.** ^1^H NMR spectrum (600 MHz) of compound **3** in CD_3_Cl

10′

11

10

6′

6

5

2

3

1

5′

11

1′

8

7′

8′

3′

7

9

4

2′

4′

9′

**Figure S20.** ^13^C NMR spectrum (150 MHz) of compound **3** in CD_3_Cl

**Figure S21.** HSQC spectrum of compound **3** in CD_3_Cl

**Figure S22.** HMBC spectrum of compound **3** in CD_3_Cl

**Figure S23.** ^1^H-^1^H COSY spectrum of compound **3** in CD_3_Cl

**Figure S24.** ROESY spectrum of compound **3** in CD_3_Cl


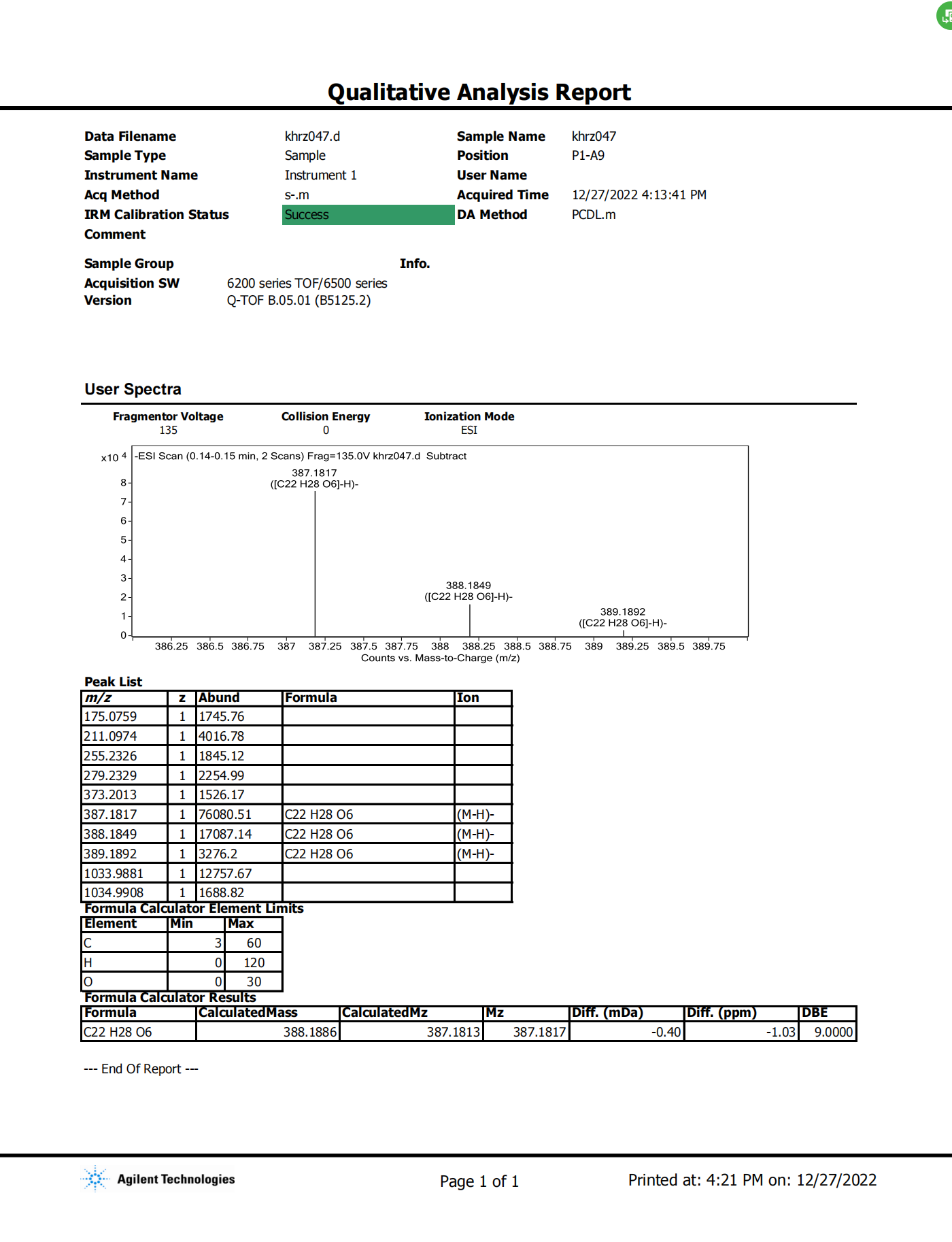


**Figure S25.** HRESI (-) MS spectrum of compound **3**


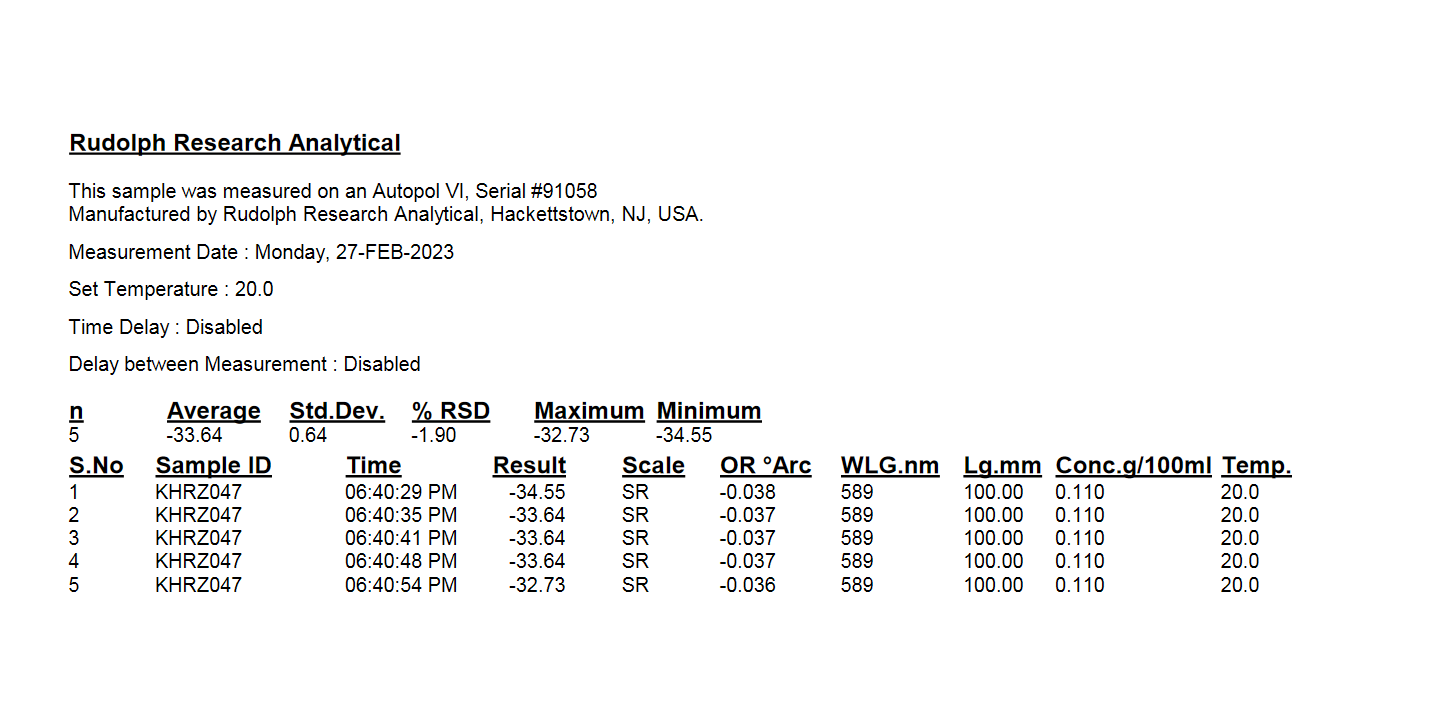


**Figure S26.** OR of compound **3**


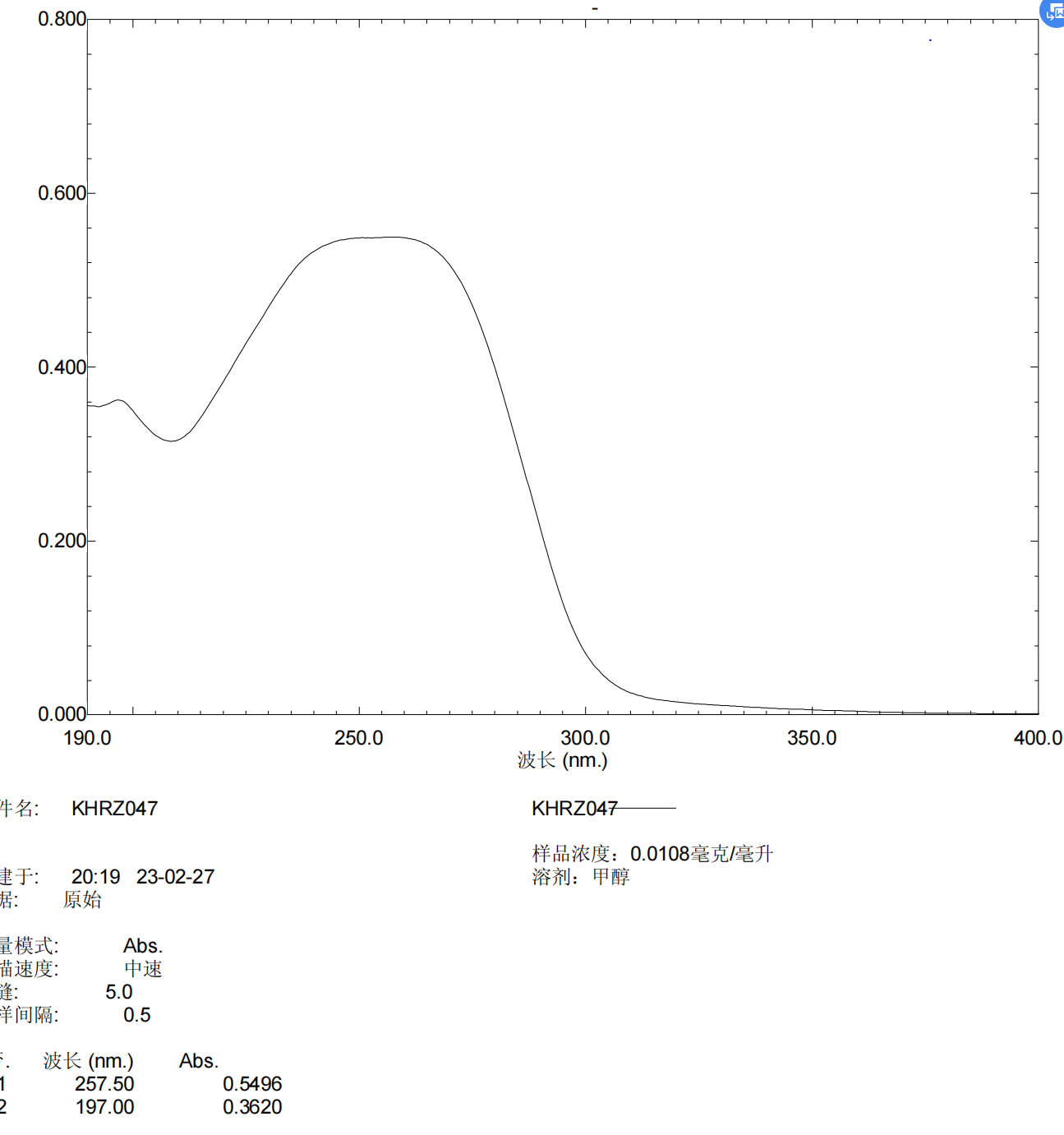


**Figure S27.** UV spectrum of compound **3**

5

6

13′,14′

1

10

6′

5′a 3

5′b

7’b

7′a

11′b

11

11′a

10′

9′

8′

1′

7

**Figure S28.** ^1^H NMR spectrum (800 MHz) of compound **4** in CD_3_Cl

10

6’

6

7’

8’

7

3’

4

9

5

2

1’

2’

13’

14’’

3

1

5’

11

11’

10’

12’

8

9’’

4’

**Figure S29.** ^13^C NMR spectrum (200 MHz) of compound **4** in CD_3_Cl

**Figure S30.** HSQC spectrum of compound **4** in CD_3_Cl

**Figure S31.** HMBC spectrum of compound **4** in CD_3_Cl

**Figure S32.** ^1^H-^1^H COSY spectrum of compound **4** in CD_3_Cl

**Figure S33.** ROESY spectrum of compound **4** in CD_3_Cl

**
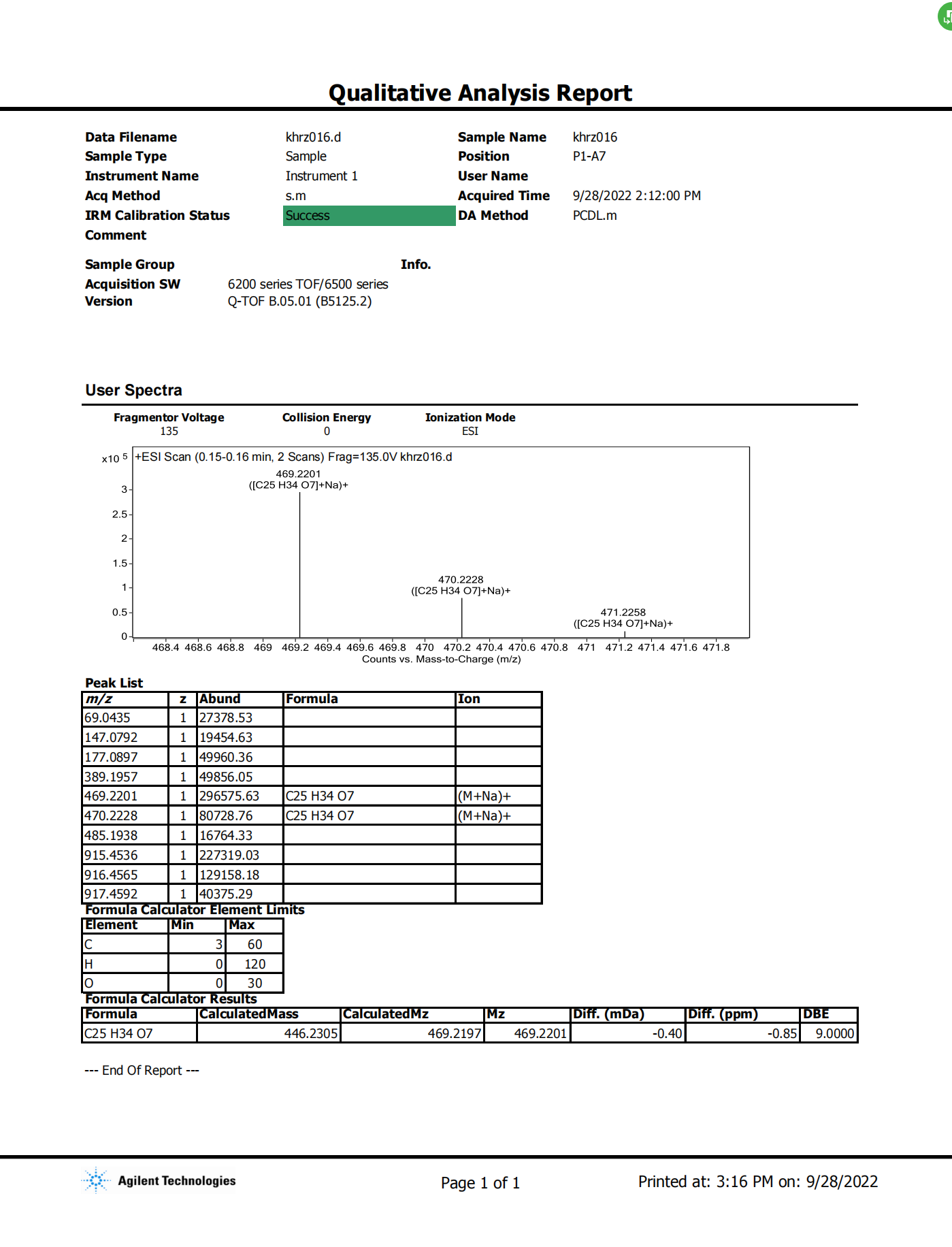
**

**Figure S34.** HRESI (+) MS spectrum of compound **4**

**
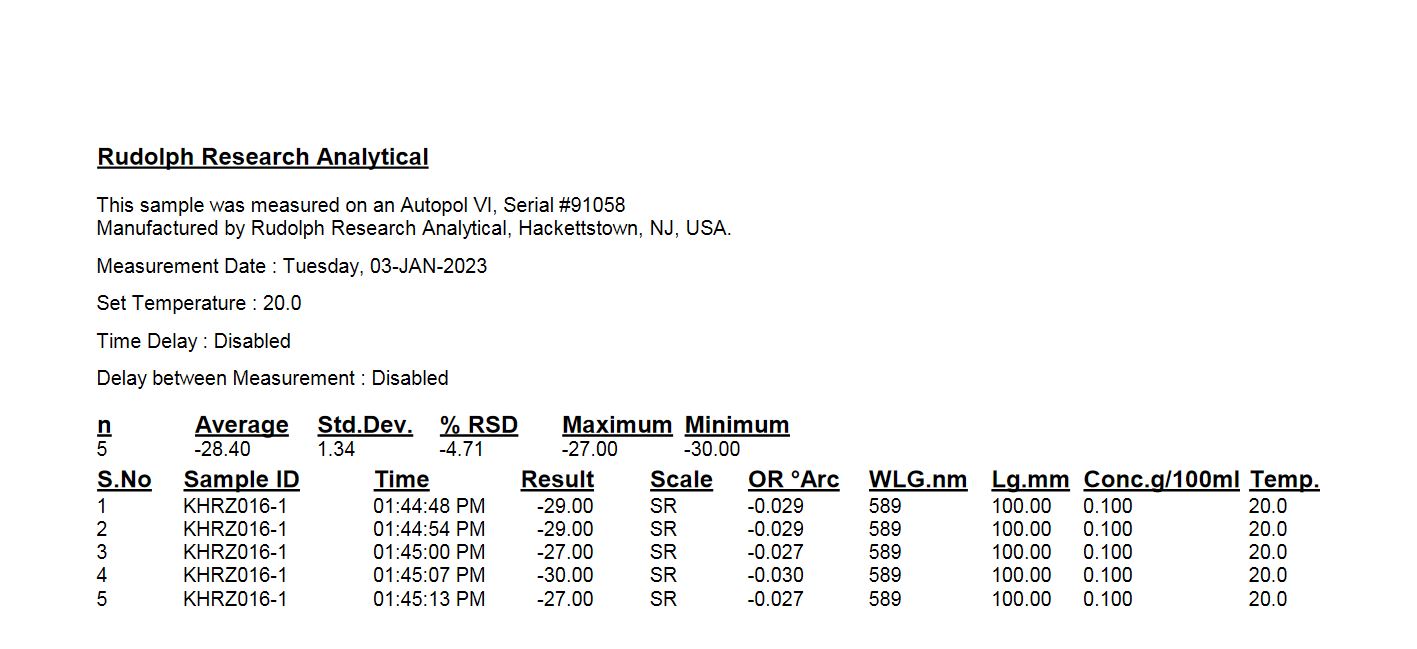
**

**Figure S35.** OR of compound **4**.


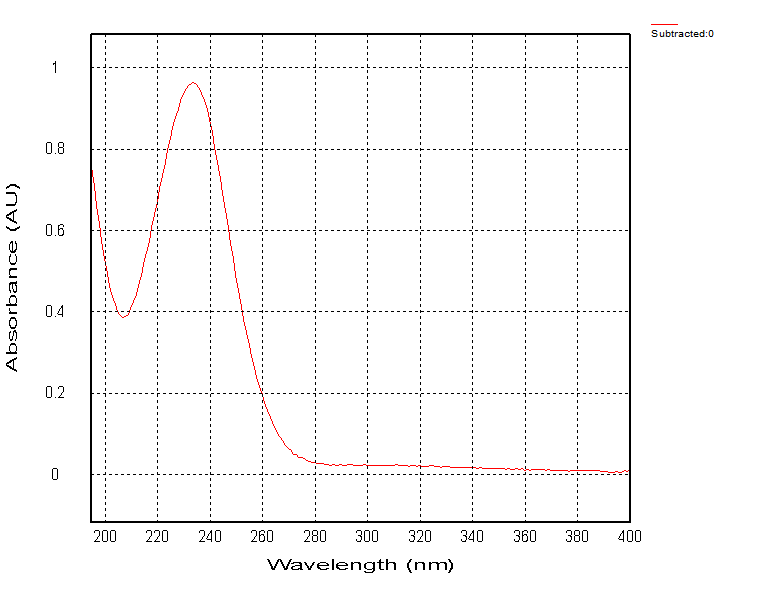


**Figure S36.** UV spectrum of compound **4**

ECD calculations: the conformers of **4** was generated within a 15 kcal/mol energy window under a MMFF94s force field via the software Gaussview 6.0.16. The obtained conformers were then imported into Gaussian 16 software and further optimized at the B3LYP/6-31G (d,p) level with SMD in MeOH. The ECD calculations were performed by using the time-dependent density functional theory (TDDFT) calculations at the B3LYP/6-31G (d,p) level with the SMD model in MeOH solution. The overall ECD curves were produced by SpecDis 1701 software on the basis of the Boltzmann weighting of each conformer [1]. The calculated ECD data as shown in the following figures and tables.

[1]Bruhn T, Schaumlöffel A, Hemberger Y, Bringmann G. SpecDis: Quantifying the comparison of calculated and experimental electronic circular dichroism spectra. Chirality. 2013; 25: 243-249.

**Figure S37.** Five optimized conformers of **4-1**

**Table S1**. Conformational analysis of the eight optimized conformers of **4-1** in the gas phase (T = 298.15 K)

| Conformer | E (Hartree) | C (Hartree) | G (kcal/mol) | ΔG (kcal/mol) | Population |
| --- | --- | --- | --- | --- | --- |
| **4**-1a | -1499.777097 | 0.482947 | -940822.072067 | 0 | 72.95% |
| **4**-1b | -1499.775435 | 0.480555 | -940822.533286 | 0.18739243 | 10.71% |
| **4**-1c | -1499.774970 | 0.482939 | -940822.742373 | 0.67030630 | 6.26% |
| **4**-1d | -1499.774862 | 0.482944 | -940822.671464 | 0.59939628 | 5.53% |
| **4**-1e | -1499.774254 | 0.483286 | -940821.075330 | 0.99673723 | 2.74% |

Electronic energy obtained at M062X/Def2TZVP SCRF=(IEFPCM, Solvent=Methanol) level of theory; Thermal correction to Gibbs free energy obtained at M062X/def2SVP SCRF=(SMD, Solvent=Methanol), Empirical Dispersion=GD3 level of theory; Gibbs free energy (E + C); The relative Gibbs free energy; The Boltzmann distribution of each conformer.

**Table S2**.Atomic coordinates (Å) of **4-1a** obtained at the CAM-B3LYP/TZVP level of theory in the MeOH.

| C | 1.891524 | 1.998511 | 0.074755 | H | -2.32113 | -0.58944 | 2.871424 |
| --- | --- | --- | --- | --- | --- | --- | --- |
| C | 2.760276 | 0.838933 | -0.17367 | H | -1.08333 | 0.145501 | 1.838318 |
| C | 3.828447 | 0.471574 | 0.561054 | H | -3.87513 | -1.90052 | 2.022914 |
| C | 4.538476 | -0.7551 | 0.108409 | H | -5.51629 | -1.57924 | 1.465291 |
| C | 6.371399 | -2.17934 | 0.495827 | H | -3.11845 | -3.58619 | -1.39526 |
| C | 4.354029 | 1.195761 | 1.772212 | H | -1.42183 | -1.661 | -2.25145 |
| C | 0.521235 | 2.0452 | -0.60478 | H | -1.62525 | -0.28928 | -1.11836 |
| C | -0.64716 | 2.408134 | 0.229594 | H | 0.477169 | -1.91856 | 1.653385 |
| C | -2.99671 | 1.927322 | 0.485801 | H | 1.431888 | -3.39416 | 1.376566 |
| C | -4.19633 | 1.929298 | -0.46114 | H | 2.052609 | -1.81486 | 0.83804 |
| C | -4.84689 | 0.556848 | -0.26174 | H | 2.284083 | -2.8807 | -1.52067 |
| C | -4.0885 | -0.16011 | 0.792635 | H | 0.80002 | -3.42447 | -2.35564 |
| C | -3.08555 | 0.616693 | 1.252438 | H | 1.487405 | -4.40058 | -1.03945 |
| C | -2.06694 | 0.306366 | 2.299607 | H | -1.83696 | -1.82497 | 0.775578 |
| C | -4.46421 | -1.56758 | 1.160805 | H | -5.2064 | -2.97149 | -0.39379 |
| C | -4.29089 | -2.53978 | 0.000644 | H | -4.91665 | 2.719335 | -0.22557 |
| C | -3.13003 | -2.87822 | -0.56877 | H | -3.88912 | 2.060953 | -1.50218 |
| C | -1.80218 | -2.29814 | -0.21238 | H | -2.93572 | 2.795539 | 1.144669 |
| C | 0.471153 | -2.61219 | -0.39244 | H | 0.325385 | 1.258256 | -1.32339 |
| C | -1.24068 | -1.30216 | -1.22736 | H | 3.2333 | 3.263456 | -2.38604 |
| C | 1.153989 | -2.4226 | 0.956608 | H | 1.624705 | 3.447255 | -3.10771 |
| C | 1.319234 | -3.37854 | -1.39388 | H | 2.233148 | 1.836272 | -2.69449 |
| C | 1.602579 | 3.044938 | -0.98878 | H | 2.492442 | 4.940611 | -0.4949 |
| C | 2.206127 | 2.880117 | -2.37148 | H | 0.891121 | 5.067668 | -1.24115 |
| C | 1.494885 | 4.486995 | -0.53265 | H | 1.035075 | 4.568908 | 0.453794 |
| H | 7.206089 | -2.23207 | 1.195263 | H | 1.882093 | 2.395986 | 1.085281 |
| H | 5.738141 | -3.06636 | 0.581938 | O | 4.203997 | -1.47887 | -0.81424 |
| H | 6.733525 | -2.10401 | -0.53285 | O | 5.638341 | -1.00157 | 0.859343 |
| H | 5.388285 | 1.520573 | 1.613674 | O | -0.62978 | 3.05158 | 1.261772 |
| H | 3.754163 | 2.077507 | 2.006701 | O | -1.77927 | 1.887613 | -0.31134 |
| H | 4.364083 | 0.540969 | 2.650655 | O | -5.82694 | 0.135702 | -0.84613 |
| H | 2.511685 | 0.207727 | -1.02211 | O | -0.77771 | -3.29671 | -0.23187 |
| H | -1.95083 | 1.149981 | 2.988416 | O | 0.147236 | -1.30229 | -0.91316 |

**Table S3.** Atomic coordinates (Å) of **4-1b** obtained at the CAM-B3LYP/TZVP level of theory in the MeOH.

| C | 1.742126 | 2.275335 | 0.126176 | H | -2.20706 | -0.76502 | 2.746232 |
| --- | --- | --- | --- | --- | --- | --- | --- |
| C | 2.66518 | 1.201728 | -0.25334 | H | -1.01951 | 0.058482 | 1.721763 |
| C | 3.50824 | 0.541342 | 0.563373 | H | -3.62538 | -2.19814 | 1.813563 |
| C | 4.30133 | -0.54099 | -0.07628 | H | -5.27591 | -2.01759 | 1.214844 |
| C | 5.748913 | -2.35809 | 0.315232 | H | -2.57227 | -3.65153 | -1.61195 |
| C | 3.673569 | 0.770919 | 2.042266 | H | -0.90866 | -1.53427 | -2.11916 |
| C | 0.385242 | 2.365388 | -0.59499 | H | -1.28734 | -0.31266 | -0.86996 |
| C | -0.81545 | 2.510671 | 0.258586 | H | 2.452203 | -2.05052 | 1.087984 |
| C | -3.09794 | 1.764164 | 0.449685 | H | 0.846241 | -2.20606 | 1.828802 |
| C | -4.28351 | 1.689426 | -0.51183 | H | 1.763462 | -3.66232 | 1.37779 |
| C | -4.80271 | 0.255898 | -0.37219 | H | 1.990372 | -4.24827 | -1.16712 |
| C | -3.98038 | -0.43141 | 0.653175 | H | 2.67204 | -2.62537 | -1.47667 |
| C | -3.05313 | 0.414179 | 1.149657 | H | 1.212992 | -3.16643 | -2.33972 |
| C | -2.0101 | 0.1533 | 2.187501 | H | -1.57002 | -2.06539 | 0.821362 |
| C | -4.22166 | -1.88473 | 0.949561 | H | -4.77283 | -3.18529 | -0.76637 |
| C | -3.91229 | -2.7639 | -0.25478 | H | -5.07708 | 2.400157 | -0.26008 |
| C | -2.69144 | -3.01486 | -0.73750 | H | -3.97755 | 1.885815 | -1.54305 |
| C | -1.42969 | -2.42031 | -0.20667 | H | -3.13879 | 2.595942 | 1.155589 |
| C | 0.871417 | -2.62231 | -0.28661 | H | 0.249783 | 1.69981 | -1.43999 |
| C | -0.83948 | -1.28862 | -1.04872 | H | 3.082257 | 3.972401 | -2.05711 |
| C | 1.52881 | -2.63358 | 1.088222 | H | 1.484283 | 4.213432 | -2.78555 |
| C | 1.748325 | -3.20322 | -1.38648 | H | 2.152141 | 2.582331 | -2.63574 |
| C | 1.428277 | 3.459217 | -0.76730 | H | 2.205856 | 5.293671 | 0.041944 |
| C | 2.072885 | 3.552541 | -2.13883 | H | 0.616087 | 5.459278 | -0.72187 |
| C | 1.233911 | 4.804985 | -0.09456 | H | 0.748353 | 4.708339 | 0.87758 |
| H | 5.083973 | -3.09717 | -0.14030 | H | 1.660861 | 2.498308 | 1.185286 |
| H | 6.45791 | -2.00802 | -0.43904 | O | 4.338165 | -0.79917 | -1.26688 |
| H | 6.272663 | -2.78996 | 1.168413 | O | 4.991 | -1.26171 | 0.843929 |
| H | 4.724909 | 0.939723 | 2.298382 | O | -0.86965 | 3.050185 | 1.347754 |
| H | 3.097799 | 1.63283 | 2.385534 | O | -1.87867 | 1.908212 | -0.33403 |
| H | 3.348546 | -0.1056 | 2.614504 | O | -5.73822 | -0.23101 | -0.97854 |
| H | 2.659458 | 0.883791 | -1.29142 | O | -0.35669 | -3.36627 | -0.24499 |
| H | -1.94446 | 0.991967 | 2.888042 | O | 0.510561 | -1.26388 | -0.60257 |

**Table S4.** Atomic coordinates (Å) of **4-1c** obtained at the CAM-B3LYP/TZVP level of theory in the MeOH.

| C | 2.233385 | 2.090365 | 0.266477 | H | -1.13412 | -0.05229 | 1.76992 |
| --- | --- | --- | --- | --- | --- | --- | --- |
| C | 3.058561 | 0.941252 | -0.11544 | H | -1.70795 | 1.336677 | 2.707076 |
| C | 3.609115 | 0.026817 | 0.707175 | H | -5.15927 | -0.87435 | 2.068448 |
| C | 4.283652 | -1.11576 | 0.030613 | H | -5.43245 | -1.42276 | 0.43234 |
| C | 5.349001 | -3.1896 | 0.35038 | H | -2.13147 | -3.60119 | 1.037601 |
| C | 3.550996 | 0.035425 | 2.211144 | H | -2.2213 | -4.21263 | -1.49538 |
| C | 0.941256 | 2.338456 | -0.54359 | H | -1.69943 | -2.91766 | -2.6136 |
| C | -0.31004 | 2.528778 | 0.227561 | H | 1.156197 | -0.49139 | -1.56374 |
| C | -2.65528 | 2.027878 | 0.183051 | H | 1.856913 | -2.05423 | -2.01149 |
| C | -3.70935 | 2.003124 | -0.92511 | H | 0.325158 | -1.51389 | -2.75197 |
| C | -4.55112 | 0.76211 | -0.62765 | H | 0.314937 | -2.80664 | 1.344159 |
| C | -4.00077 | 0.113729 | 0.582311 | H | 1.270347 | -1.36278 | 0.924369 |
| C | -2.92201 | 0.792562 | 1.026528 | H | 1.815122 | -2.96753 | 0.396806 |
| C | -2.02967 | 0.441343 | 2.168021 | H | -2.97106 | -1.54716 | -1.09931 |
| C | -4.64017 | -1.13026 | 1.135347 | H | -3.94976 | -2.84997 | 2.329963 |
| C | -3.72351 | -2.30459 | 1.414625 | H | -4.34187 | 2.896028 | -0.94229 |
| C | -2.69942 | -2.74415 | 0.677866 | H | -3.23942 | 1.910042 | -1.90838 |
| C | -2.21371 | -2.17527 | -0.62688 | H | -2.68234 | 2.92969 | 0.800548 |
| C | 0.127367 | -2.17498 | -0.70466 | H | 0.812758 | 1.736919 | -1.43639 |
| C | -1.68772 | -3.26216 | -1.56932 | H | 3.865339 | 3.769941 | -1.71853 |
| C | 0.918609 | -1.52248 | -1.83297 | H | 2.35021 | 4.20019 | -2.53155 |
| C | 0.935071 | -2.34165 | 0.573784 | H | 2.856971 | 2.509403 | -2.44635 |
| C | 2.083366 | 3.340325 | -0.57439 | H | 1.444882 | 5.399586 | -0.46601 |
| C | 2.830897 | 3.451163 | -1.89193 | H | 1.396753 | 4.557673 | 1.090792 |
| C | 1.958527 | 4.661423 | 0.161881 | H | 2.957135 | 5.052728 | 0.389546 |
| H | 6.22037 | -2.90265 | -0.24378 | H | 2.086828 | 2.257253 | 1.329095 |
| H | 5.645363 | -3.8117 | 1.195199 | O | 4.412476 | -1.25903 | -1.1725 |
| H | 4.643897 | -3.72311 | -0.29285 | O | 4.726804 | -2.0318 | 0.924625 |
| H | 4.558366 | 0.002081 | 2.639982 | O | -0.4122 | 3.08488 | 1.306558 |
| H | 3.046856 | 0.925505 | 2.593114 | O | -1.34255 | 1.962665 | -0.43385 |
| H | 3.022036 | -0.84674 | 2.588753 | O | -5.49962 | 0.36782 | -1.28378 |
| H | 3.206947 | 0.769149 | -1.17771 | O | -1.03541 | -1.36543 | -0.44581 |
| H | -2.50958 | -0.25915 | 2.855345 | O | -0.36146 | -3.45889 | -1.09809 |

**Table S5.** Atomic coordinates (Å) of **4-1d** obtained at the CAM-B3LYP/TZVP level of theory in the MeOH.

| C | -2.44271 | 2.135714 | -0.39519 | H | 2.251987 | 0.032182 | -2.86825 |
| --- | --- | --- | --- | --- | --- | --- | --- |
| C | -3.2661 | 0.930406 | -0.17728 | H | 0.904837 | 0.129435 | -1.7362 |
| C | -3.127 | -0.2268 | -0.85211 | H | 4.991389 | -0.43007 | -2.21997 |
| C | -4.05205 | -1.32661 | -0.45649 | H | 5.34698 | -1.08619 | -0.64345 |
| C | -4.62797 | -3.58327 | -0.80763 | H | 2.190344 | -3.45001 | -1.46183 |
| C | -2.08212 | -0.49293 | -1.90238 | H | 1.93317 | -4.19852 | 0.954475 |
| C | -1.23266 | 2.393604 | 0.532762 | H | 2.964507 | -3.15479 | 1.978193 |
| C | 0.051969 | 2.672679 | -0.14238 | H | -1.45334 | -0.65162 | 1.334747 |
| C | 2.393306 | 2.172436 | -0.07428 | H | -1.00641 | -1.26808 | 2.942189 |
| C | 3.438693 | 2.086469 | 1.038041 | H | 0.148467 | -0.27461 | 2.015182 |
| C | 4.3791 | 0.965741 | 0.591822 | H | -1.79167 | -3.00525 | 0.227575 |
| C | 3.817226 | 0.359999 | -0.63734 | H | -1.65854 | -3.60796 | 1.898397 |
| C | 2.689591 | 1.006535 | -1.00146 | H | -0.60505 | -4.2631 | 0.622248 |
| C | 1.771334 | 0.677059 | -2.12976 | H | 2.473307 | -1.20514 | 0.635433 |
| C | 4.514993 | -0.79534 | -1.29995 | H | 4.027457 | -2.51533 | -2.56999 |
| C | 3.692438 | -2.01446 | -1.66258 | H | 3.990584 | 3.016632 | 1.199984 |
| C | 2.652814 | -2.56459 | -1.02687 | H | 2.966671 | 1.810885 | 1.986615 |
| C | 2.032413 | -2.13197 | 0.259687 | H | 2.420178 | 3.115097 | -0.62713 |
| C | -0.05141 | -2.29128 | 1.30026 | H | -1.14816 | 1.749863 | 1.399582 |
| C | 2.05863 | -3.18611 | 1.368662 | H | -4.32087 | 3.589938 | 1.538458 |
| C | -0.63019 | -1.03889 | 1.940488 | H | -2.89741 | 4.018919 | 2.502816 |
| C | -1.09629 | -3.35846 | 0.993045 | H | -3.30622 | 2.318375 | 2.240862 |
| C | -2.43601 | 3.332633 | 0.511171 | H | -1.89644 | 5.422457 | 0.600017 |
| C | -3.28731 | 3.305045 | 1.768605 | H | -1.68726 | 4.700271 | -1.00295 |
| C | -2.32192 | 4.70944 | -0.11628 | H | -3.3191 | 5.070053 | -0.39552 |
| H | -4.48364 | -3.85325 | 0.241802 | H | -2.19567 | 2.358225 | -1.43074 |
| H | -5.68219 | -3.34592 | -0.97105 | O | -4.92331 | -1.25634 | 0.389497 |
| H | -4.308 | -4.39524 | -1.46083 | O | -3.80126 | -2.46321 | -1.154 |
| H | -1.31244 | -1.17396 | -1.51876 | O | 0.203065 | 3.324908 | -1.15994 |
| H | -2.51871 | -0.96511 | -2.78741 | O | 1.067882 | 2.048134 | 0.505527 |
| H | -1.58288 | 0.429203 | -2.20883 | O | 5.401926 | 0.622534 | 1.156945 |
| H | -4.03453 | 0.960997 | 0.590575 | O | 0.617541 | -1.95385 | 0.069893 |
| H | 1.397982 | 1.586145 | -2.60954 | O | 0.955162 | -2.80803 | 2.17558 |

**Table S6.** Atomic coordinates (Å) of **4-1e** obtained at the CAM-B3LYP/TZVP level of theory in the MeOH.

| C | 2.671262 | 1.821684 | 0.478216 | H | -1.5766 | 2.566154 | 2.767569 |
| --- | --- | --- | --- | --- | --- | --- | --- |
| C | 3.316219 | 0.492054 | 0.377064 | H | -2.35647 | 1.031595 | 3.259306 |
| C | 2.829448 | -0.62017 | 0.956625 | H | -4.59149 | -0.11185 | 2.464739 |
| C | 3.556911 | -1.88689 | 0.664406 | H | -4.77058 | -1.03571 | 0.982874 |
| C | 3.482147 | -4.23469 | 0.818639 | H | -1.39788 | -2.83781 | 1.875587 |
| C | 1.565878 | -0.66255 | 1.772992 | H | -0.94413 | -3.98107 | -0.23125 |
| C | 1.51439 | 2.143256 | -0.4895 | H | -2.54863 | -4.0636 | -1.02115 |
| C | 0.263964 | 2.657433 | 0.108211 | H | -2.19685 | -0.64767 | -2.76657 |
| C | -2.12323 | 2.404932 | 0.015026 | H | -0.6881 | 0.29401 | -2.68116 |
| C | -3.12903 | 2.082613 | -1.09151 | H | -0.84984 | -1.03616 | -3.85926 |
| C | -3.83909 | 0.820983 | -0.60383 | H | 1.297488 | -2.12808 | -2.8124 |
| C | -3.44506 | 0.596031 | 0.803599 | H | 1.273996 | -2.52709 | -1.07592 |
| C | -2.48325 | 1.471019 | 1.16091 | H | 1.426075 | -0.83964 | -1.59167 |
| C | -1.78135 | 1.532783 | 2.47576 | H | -2.99589 | -1.72957 | -0.51853 |
| C | -4.03371 | -0.5272 | 1.616932 | H | -2.96679 | -1.64262 | 3.220807 |
| C | -3.0022 | -1.51168 | 2.140898 | H | -3.8668 | 2.882733 | -1.21891 |
| C | -2.13002 | -2.19255 | 1.39231 | H | -2.64444 | 1.916343 | -2.0554 |
| C | -2.06138 | -2.11892 | -0.09944 | H | -2.1135 | 3.450452 | 0.331998 |
| C | -0.56068 | -1.67517 | -1.81221 | H | 1.355106 | 1.427057 | -1.28733 |
| C | -1.69218 | -3.41764 | -0.81166 | H | 3.392607 | 3.310179 | -2.58381 |
| C | -1.10754 | -0.70219 | -2.84943 | H | 3.588421 | 1.612434 | -2.12139 |
| C | 0.956596 | -1.79841 | -1.82657 | H | 4.744541 | 2.824858 | -1.54623 |
| C | 2.834664 | 2.912571 | -0.53717 | H | 2.250687 | 4.513231 | 0.81055 |
| C | 3.685591 | 2.642664 | -1.76559 | H | 3.926766 | 4.604209 | 0.216796 |
| C | 2.895095 | 4.349327 | -0.05391 | H | 2.578409 | 5.035028 | -0.84895 |
| H | 4.468145 | -4.29542 | 1.286506 | H | 2.45725 | 2.17058 | 1.487504 |
| H | 2.810941 | -4.9868 | 1.234152 | O | 4.621229 | -1.97959 | 0.08316 |
| H | 3.594025 | -4.3716 | -0.26015 | O | 2.866739 | -2.97061 | 1.102599 |
| H | 0.713024 | -0.9625 | 1.151205 | O | 0.171893 | 3.438699 | 1.038514 |
| H | 1.646801 | -1.37998 | 2.593524 | O | -0.8017 | 2.086682 | -0.5007 |
| H | 1.342027 | 0.321871 | 2.193137 | O | -4.59088 | 0.120196 | -1.26044 |
| H | 4.215453 | 0.391676 | -0.22508 | O | -0.96722 | -1.2698 | -0.49688 |
| H | -0.81169 | 1.025425 | 2.403873 | O | -1.1579 | -2.96015 | -2.0437 |

**Figure S38.** Seven optimized conformers of **4-2**

**Table S7.** Conformational analysis of the eight optimized conformers of **4-2** in the gas phase (T = 298.15 K)

| Conformer | E (Hartree) | C (Hartree) | G (kcal/mol) | ΔG (kcal/mol) | Population |
| --- | --- | --- | --- | --- | --- |
| **4**-2a | -1499.777340 | 0.481998 | -940822.820058 | 0 | 43.03% |
| **4**-2b | -1499.776987 | 0.481988 | -940822.604822 | 0.21523593 | 28.63% |
| **4**-2c | -1499.776274 | 0.481462 | -940822.487478 | 0.33258030 | 12.57% |
| **4**-2d | -1499.775437 | 0.481284 | -940822.073949 | 0.74610939 | 4.78% |
| **4**-2e | -1499.775360 | 0.481485 | -940821.899501 | 0.92055717 | 4.37% |
| **4**-2f | -1499.774314 | 0.480830 | -940821.654145 | 1.16591358 | 4.22% |
| **4**-2g | -1499.773608 | 0.480308 | -940821.538683 | 1.28137542 | 1.31% |

Electronic energy obtained at M062X/Def2TZVP SCRF=(IEFPCM, Solvent=Methanol) level of theory; Thermal correction to Gibbs free energy obtained at M062X/def2SVP SCRF=(SMD, Solvent=Methanol), Empirical Dispersion=GD3 level of theory; Gibbs free energy (E + C); The relative Gibbs free energy; The Boltzmann distribution of each conformer.e

**Table S8.** Atomic coordinates (Å) of **4-2a** obtained at the CAM-B3LYP/TZVP level of theory in the MeOH.

| C | -3.21964 | 1.031088 | 0.324991 | H | 2.590361 | 0.14711 | 3.168231 |
| --- | --- | --- | --- | --- | --- | --- | --- |
| C | -4.40169 | 0.205716 | 0.047414 | H | 2.759678 | 0.876918 | -2.93124 |
| C | -4.93337 | 0.026738 | -1.17753 | H | 1.669551 | 1.807264 | -1.86791 |
| C | -7.74706 | -2.19772 | -0.31431 | H | 2.765962 | 2.655683 | -2.98718 |
| C | -6.13587 | -0.83155 | -1.34984 | H | 2.674902 | 3.033308 | 0.173961 |
| C | -4.38424 | 0.650587 | -2.43858 | H | 3.733013 | 3.942288 | -0.93145 |
| C | -3.03143 | 1.814638 | 1.608516 | H | 4.437322 | 3.00045 | 0.405778 |
| C | -2.19605 | 0.562654 | 1.373692 | H | 6.600886 | 0.436611 | -1.70299 |
| C | -0.764 | 0.621523 | 0.997046 | H | 6.247551 | 1.371517 | -0.22028 |
| C | 1.359383 | -0.37369 | 1.401248 | H | 5.036013 | -0.61468 | 0.367687 |
| C | 1.711063 | -1.45268 | 0.401428 | H | 4.784121 | -1.01932 | -2.65756 |
| C | 2.82347 | -2.11395 | 0.782993 | H | 3.999556 | -3.2053 | -2.20833 |
| C | 3.249405 | -1.64698 | 2.118314 | H | 3.13616 | -4.00948 | -0.17559 |
| C | 2.160213 | -0.71615 | 2.655396 | H | 4.548863 | -3.31021 | 0.592415 |
| C | 3.670238 | -3.06429 | -0.01943 | H | 1.299091 | -2.42619 | -1.47155 |
| C | 4.097801 | -2.5169 | -1.37009 | H | 1.113029 | -0.65977 | -1.47279 |
| C | 4.555991 | -1.28695 | -1.62662 | H | -0.11116 | -1.66624 | -0.69537 |
| C | 4.783613 | -0.19703 | -0.60965 | H | 1.663719 | 0.584405 | 0.977719 |
| C | 5.803661 | 0.843836 | -1.07662 | H | -2.46225 | -0.30374 | 1.970056 |
| C | 3.731666 | 1.801358 | -1.25097 | H | -4.91396 | 2.394901 | 2.495476 |
| C | 2.660602 | 1.785643 | -2.33148 | H | -4.48092 | 0.708616 | 2.818417 |
| C | 3.639884 | 3.024573 | -0.34278 | H | -3.63961 | 2.011592 | 3.666369 |
| C | 0.965183 | -1.5705 | -0.88105 | H | -1.91936 | 3.463516 | 2.451053 |
| C | -4.07719 | 1.718285 | 2.705166 | H | -3.16428 | 3.935531 | 1.28226 |
| C | -2.3936 | 3.187069 | 1.501231 | H | -1.63513 | 3.223429 | 0.718213 |
| H | -7.53908 | -3.04892 | -0.96825 | H | -2.74402 | 1.485912 | -0.5385 |
| H | -7.97594 | -2.53744 | 0.696398 | O | -6.67629 | -1.05009 | -2.41851 |
| H | -8.58591 | -1.63239 | -0.7292 | O | -6.58927 | -1.36354 | -0.18628 |
| H | -4.40735 | 1.745397 | -2.38687 | O | -0.27484 | 1.385836 | 0.183407 |
| H | -4.97924 | 0.33633 | -3.29694 | O | -0.05942 | -0.31287 | 1.677765 |
| H | -3.34356 | 0.353007 | -2.61206 | O | 4.298654 | -1.9245 | 2.675935 |
| H | -4.87191 | -0.2937 | 0.887599 | O | 5.013451 | 1.715796 | -1.87548 |
| H | 1.531097 | -1.26282 | 3.367322 | O | 3.614531 | 0.629932 | -0.42632 |

**Table S9.** Atomic coordinates (Å) of **4-2b** obtained at the CAM-B3LYP/TZVP level of theory in the MeOH.

| C | 3.435835 | 0.781597 | 0.77546 | H | -2.24018 | 1.96389 | 1.581873 |
| --- | --- | --- | --- | --- | --- | --- | --- |
| C | 4.546738 | 0.278535 | -0.04018 | H | -3.2492 | -3.95422 | 1.018576 |
| C | 5.258017 | -0.84642 | 0.167855 | H | -1.7948 | -3.02383 | 1.447525 |
| C | 6.337392 | -1.11688 | -0.82386 | H | -2.91848 | -3.55202 | 2.722096 |
| C | 8.05881 | -2.5969 | -1.44373 | H | -2.11605 | -0.59626 | 2.375222 |
| C | 5.058717 | -1.82814 | 1.291708 | H | -3.3901 | -1.08531 | 3.514223 |
| C | 3.19296 | 2.259182 | 1.013944 | H | -3.74654 | 0.124183 | 2.268051 |
| C | 2.260114 | 1.490165 | 0.085055 | H | -5.79262 | -3.02147 | 0.039622 |
| C | 0.890714 | 1.100176 | 0.493745 | H | -6.72147 | -1.64788 | 0.708933 |
| C | -1.36553 | 1.002872 | -0.21195 | H | -5.03268 | -0.05764 | -0.00297 |
| C | -2.11925 | 0.754557 | -1.49432 | H | -5.14899 | -2.28722 | -2.1166 |
| C | -3.33695 | 1.334496 | -1.45981 | H | -4.96651 | -0.5662 | -3.73267 |
| C | -3.49221 | 2.100435 | -0.20381 | H | -4.26921 | 1.650072 | -3.36567 |
| C | -2.13896 | 2.110483 | 0.505667 | H | -5.34932 | 1.681761 | -1.9843 |
| C | -4.48713 | 1.153124 | -2.41251 | H | -2.10409 | -0.11487 | -3.46536 |
| C | -4.83084 | -0.29978 | -2.68575 | H | -1.67586 | -1.21008 | -2.14177 |
| C | -4.95011 | -1.27196 | -1.77605 | H | -0.50558 | 0.007232 | -2.6813 |
| C | -4.83481 | -1.09635 | -0.29248 | H | -1.35879 | 0.082172 | 0.375697 |
| C | -5.71604 | -2.03749 | 0.528633 | H | 2.364779 | 1.704758 | -0.97333 |
| C | -3.63864 | -1.89944 | 1.531668 | H | 3.5873 | 4.256367 | 0.308432 |
| C | -2.84862 | -3.19108 | 1.691737 | H | 5.016794 | 3.41356 | 0.930923 |
| C | -3.18792 | -0.79237 | 2.479422 | H | 4.374462 | 3.010241 | -0.66817 |
| C | -1.57088 | -0.18184 | -2.5142 | H | 2.055353 | 1.928517 | 2.837988 |
| C | 4.094711 | 3.285735 | 0.351862 | H | 2.178218 | 3.626073 | 2.343309 |
| C | 2.718374 | 2.672598 | 2.394318 | H | 3.582433 | 2.813482 | 3.054321 |
| H | 8.481032 | -3.5308 | -1.07156 | H | 3.107731 | 0.150546 | 1.595429 |
| H | 7.668361 | -2.72642 | -2.45668 | O | 6.617246 | -0.42162 | -1.78335 |
| H | 8.816641 | -1.809 | -1.45732 | O | 7.001377 | -2.26302 | -0.53519 |
| H | 4.241378 | -1.53119 | 1.951918 | O | 0.573722 | 0.61678 | 1.56522 |
| H | 5.968768 | -1.92194 | 1.894529 | O | 0.006433 | 1.353929 | -0.50112 |
| H | 4.838646 | -2.8284 | 0.902821 | O | -4.52812 | 2.593454 | 0.211207 |
| H | 4.83335 | 0.866834 | -0.90784 | O | -5.03479 | -2.12993 | 1.76865 |
| H | -1.64765 | 3.075357 | 0.334146 | O | -3.52263 | -1.48488 | 0.16276 |

**Table S10.** Atomic coordinates (Å) of **4-2c** obtained at the CAM-B3LYP/TZVP level of theory in the MeOH.

| C | 3.235482 | 1.147052 | 0.153703 | H | -2.76717 | 2.048924 | -2.22423 |
| --- | --- | --- | --- | --- | --- | --- | --- |
| C | 4.399828 | 0.3026 | -0.13402 | H | -3.7279 | 1.269625 | 4.246497 |
| C | 5.037475 | -0.51508 | 0.726077 | H | -3.91837 | -0.46189 | 3.878999 |
| C | 6.198112 | -1.26153 | 0.162686 | H | -2.35178 | 0.330243 | 3.616928 |
| C | 7.905648 | -2.81089 | 0.633453 | H | -3.59272 | 2.952122 | 2.272979 |
| C | 4.683947 | -0.73193 | 2.173352 | H | -3.59 | 2.312407 | 0.616783 |
| C | 3.017318 | 2.501191 | -0.49165 | H | -2.1626 | 2.080686 | 1.664141 |
| C | 2.152103 | 1.323525 | -0.92368 | H | -5.84741 | -1.07583 | 2.414054 |
| C | 0.740738 | 1.176914 | -0.49718 | H | -6.67728 | -0.24827 | 1.064831 |
| C | -1.43142 | 0.560075 | -1.27169 | H | -4.72397 | -0.39509 | -0.34643 |
| C | -1.76346 | -0.91215 | -1.15964 | H | -4.6578 | -2.87884 | 1.465005 |
| C | -2.87269 | -1.21191 | -1.86757 | H | -4.03119 | -4.11171 | -0.43696 |
| C | -3.33591 | -0.01995 | -2.60979 | H | -3.09356 | -3.27001 | -2.41257 |
| C | -2.29331 | 1.083432 | -2.4191 | H | -4.55416 | -2.30926 | -2.50779 |
| C | -3.66321 | -2.49055 | -1.89059 | H | 0.09252 | -1.69564 | -0.44544 |
| C | -4.06642 | -3.02719 | -0.53083 | H | -1.27128 | -2.83597 | -0.33167 |
| C | -4.44383 | -2.33221 | 0.547078 | H | -1.15679 | -1.46467 | 0.789262 |
| C | -4.59286 | -0.84375 | 0.644323 | H | -1.66555 | 1.041022 | -0.32198 |
| C | -5.71844 | -0.37822 | 1.571543 | H | 2.363246 | 0.916355 | -1.90685 |
| C | -3.84727 | 0.811907 | 2.133212 | H | 3.538497 | 3.780271 | -2.14505 |
| C | -3.43599 | 0.467448 | 3.561874 | H | 4.876533 | 3.461883 | -1.02698 |
| C | -3.25543 | 2.124417 | 1.641352 | H | 4.373795 | 2.222698 | -2.18652 |
| C | -0.98106 | -1.78699 | -0.24412 | H | 3.233292 | 4.106696 | 0.921664 |
| C | 4.009022 | 3.010542 | -1.52255 | H | 1.706884 | 3.209796 | 1.092678 |
| C | 2.429807 | 3.599395 | 0.374725 | H | 1.924963 | 4.348321 | -0.24788 |
| H | 7.613589 | -3.47602 | -0.1836 | H | 2.81003 | 1.068368 | 1.149191 |
| H | 8.695195 | -2.14339 | 0.278015 | O | 6.603508 | -1.19352 | -0.98326 |
| H | 8.246459 | -3.38591 | 1.494929 | O | 6.77809 | -2.05584 | 1.09576 |
| H | 5.523911 | -0.46672 | 2.824883 | O | 0.313561 | 1.375353 | 0.626152 |
| H | 4.460846 | -1.78683 | 2.367365 | O | -0.02448 | 0.772259 | -1.53908 |
| H | 3.818012 | -0.13907 | 2.474712 | O | -4.37608 | 0.074989 | -3.23878 |
| H | 4.796468 | 0.329089 | -1.14543 | O | -5.26718 | 0.892109 | 2.01196 |
| H | -1.69177 | 1.177592 | -3.33009 | O | -3.43267 | -0.26884 | 1.277323 |

**Table S11.** Atomic coordinates (Å) of **4-2d** obtained at the CAM-B3LYP/TZVP level of theory in the MeOH.

| C | -3.20954 | 1.023099 | 0.34637 | H | 2.579066 | -0.03633 | 3.16412 |
| --- | --- | --- | --- | --- | --- | --- | --- |
| C | -4.39701 | 0.22046 | 0.024963 | H | 1.701572 | 1.904498 | -1.77274 |
| C | -4.98145 | 0.105942 | -1.18383 | H | 2.807607 | 2.815837 | -2.83135 |
| C | -6.18585 | -0.75101 | -1.3478 | H | 2.804885 | 1.036706 | -2.87455 |
| C | -7.75032 | -2.18718 | -0.3361 | H | 2.682357 | 3.014397 | 0.346426 |
| C | -4.53689 | 0.778211 | -2.45503 | H | 3.747603 | 3.987266 | -0.69574 |
| C | -3.02186 | 1.726486 | 1.675476 | H | 4.442648 | 2.973693 | 0.592953 |
| C | -2.19158 | 0.486181 | 1.366055 | H | 6.633535 | 0.535924 | -1.63749 |
| C | -0.7578 | 0.562268 | 1.000193 | H | 6.264022 | 1.381702 | -0.10595 |
| C | 1.362558 | -0.45616 | 1.360711 | H | 5.050454 | -0.63748 | 0.352012 |
| C | 1.723636 | -1.47572 | 0.303748 | H | 4.828237 | -0.86275 | -2.69397 |
| C | 2.833477 | -2.15731 | 0.656087 | H | 4.039853 | -3.0711 | -2.38237 |
| C | 3.247711 | -1.76749 | 2.019509 | H | 3.155653 | -3.99331 | -0.40953 |
| C | 2.153576 | -0.86921 | 2.599751 | H | 4.560556 | -3.34138 | 0.412673 |
| C | 3.688152 | -3.05934 | -0.19241 | H | 1.136412 | -0.5756 | -1.52492 |
| C | 4.129466 | -2.43339 | -1.50403 | H | -0.08915 | -1.63068 | -0.81823 |
| C | 4.589905 | -1.19059 | -1.68295 | H | 1.330184 | -2.33834 | -1.62667 |
| C | 4.806718 | -0.16268 | -0.60109 | H | 1.669315 | 0.524819 | 0.995284 |
| C | 5.829499 | 0.904765 | -0.9962 | H | -2.4633 | -0.41301 | 1.909044 |
| C | 3.757029 | 1.867794 | -1.13519 | H | -4.90544 | 2.258452 | 2.58998 |
| C | 2.697463 | 1.91045 | -2.22628 | H | -4.47521 | 0.556471 | 2.81987 |
| C | 3.652203 | 3.037589 | -0.16062 | H | -3.63366 | 1.809164 | 3.739879 |
| C | 0.9882 | -1.52033 | -0.98932 | H | -1.90615 | 3.316676 | 2.620051 |
| C | -4.06985 | 1.570149 | 2.76317 | H | -3.1432 | 3.863643 | 1.475889 |
| C | -2.37705 | 3.099687 | 1.6533 | H | -1.6146 | 3.179312 | 0.87737 |
| H | -8.60624 | -1.60205 | -0.68312 | H | -2.72604 | 1.52915 | -0.48285 |
| H | -7.56504 | -2.99542 | -1.04874 | O | -6.76399 | -0.90383 | -2.40778 |
| H | -7.93872 | -2.58936 | 0.65989 | O | -6.59092 | -1.35583 | -0.20213 |
| H | -5.33749 | 1.41007 | -2.85494 | O | -0.26258 | 1.371724 | 0.235273 |
| H | -4.3202 | 0.032433 | -3.22751 | O | -0.0585 | -0.41169 | 1.629075 |
| H | -3.64869 | 1.395075 | -2.30889 | O | 4.292387 | -2.07627 | 2.569225 |
| H | -4.84266 | -0.33519 | 0.843477 | O | 5.04577 | 1.821287 | -1.75017 |
| H | 1.519094 | -1.45549 | 3.274371 | O | 3.635363 | 0.65162 | -0.37908 |

**Table S12.** Atomic coordinates (Å) of **4-2e** obtained at the CAM-B3LYP/TZVP level of theory in the MeOH.

| C | -2.76323 | -1.69849 | 0.145819 | H | 3.614362 | -3.74069 | -0.68035 |
| --- | --- | --- | --- | --- | --- | --- | --- |
| C | -3.32883 | -0.36113 | -0.06869 | H | 1.141688 | 0.182854 | -1.33608 |
| C | -3.83282 | 0.455084 | 0.877132 | H | 0.293494 | 1.368564 | -2.36267 |
| C | -4.27943 | 1.82483 | 0.510114 | H | 2.043478 | 1.512587 | -2.07739 |
| C | -4.40742 | 3.486568 | -1.1539 | H | -0.54314 | 0.681506 | 0.530348 |
| C | -3.9837 | 0.134991 | 2.339696 | H | -1.34443 | 1.965092 | -0.38877 |
| C | -2.81759 | -2.81799 | -0.87112 | H | -0.62624 | 2.331556 | 1.197383 |
| C | -1.50338 | -2.0829 | -0.65932 | H | 2.546639 | 3.897898 | -1.71745 |
| C | -0.36346 | -2.62903 | 0.113022 | H | 2.110936 | 4.97907 | -0.37188 |
| C | 2.035304 | -2.5309 | 0.268393 | H | 3.065898 | 3.486431 | 1.209393 |
| C | 2.446629 | -1.31057 | 1.07438 | H | 4.541171 | 3.013532 | -1.15656 |
| C | 3.631181 | -0.81642 | 0.658552 | H | 5.654959 | 1.026561 | -0.52136 |
| C | 4.183535 | -1.66259 | -0.4243 | H | 5.296844 | 0.046832 | 1.654176 |
| C | 3.155531 | -2.74977 | -0.74721 | H | 3.77603 | 0.913087 | 1.904629 |
| C | 4.377605 | 0.389628 | 1.158986 | H | 1.892215 | 0.15195 | 2.554477 |
| C | 4.771117 | 1.32292 | 0.035887 | H | 1.569428 | -1.54614 | 3.005178 |
| C | 4.122278 | 2.428101 | -0.33775 | H | 0.535199 | -0.72869 | 1.849065 |
| C | 2.848504 | 2.97977 | 0.260059 | H | 1.837346 | -3.39448 | 0.90692 |
| C | 2.106106 | 3.939633 | -0.71101 | H | -1.21087 | -1.39745 | -1.44767 |
| C | 0.802234 | 2.097727 | -0.3881 | H | -3.31575 | -1.58267 | -2.60727 |
| C | 1.088451 | 1.23686 | -1.62116 | H | -3.13903 | -3.30888 | -2.94521 |
| C | -0.51114 | 1.744498 | 0.282767 | H | -4.58414 | -2.71327 | -2.10924 |
| C | 1.573509 | -0.82119 | 2.180563 | H | -4.05593 | -4.44273 | -0.19833 |
| C | -3.50005 | -2.58335 | -2.20732 | H | -2.46889 | -4.37568 | 0.604569 |
| C | -2.99014 | -4.22979 | -0.34241 | H | -2.59774 | -4.95705 | -1.0636 |
| H | -5.48569 | 3.617109 | -1.03015 | H | -2.71323 | -2.04619 | 1.172571 |
| H | -3.88755 | 4.226962 | -0.54066 | O | -4.8127 | 2.592783 | 1.288275 |
| H | -4.12439 | 3.590119 | -2.20171 | O | -4.01281 | 2.159553 | -0.77942 |
| H | -5.01411 | 0.313692 | 2.663069 | O | -0.43386 | -3.30463 | 1.122461 |
| H | -3.35144 | 0.793742 | 2.946336 | O | 0.803718 | -2.22354 | -0.44429 |
| H | -3.72163 | -0.89995 | 2.566063 | O | 5.273666 | -1.53916 | -0.95416 |
| H | -3.29357 | 0.020661 | -1.0836 | O | 0.765689 | 3.48328 | -0.71827 |
| H | 2.788845 | -2.62227 | -1.77019 | O | 1.866853 | 1.976383 | 0.561312 |

**Table S13.** Atomic coordinates (Å) of **4-2f** obtained at the CAM-B3LYP/TZVP level of theory in the MeOH.

| C | -3.19595 | 1.139674 | -0.11645 | H | 2.739672 | 1.833749 | 2.514438 |
| --- | --- | --- | --- | --- | --- | --- | --- |
| C | -4.38485 | 0.32414 | 0.153523 | H | 2.67298 | -0.86625 | -2.96263 |
| C | -5.02841 | -0.479 | -0.71583 | H | 1.637123 | 0.521788 | -2.53321 |
| C | -6.21562 | -1.19638 | -0.1703 | H | 2.683838 | 0.598681 | -3.97311 |
| C | -7.95236 | -2.70477 | -0.66671 | H | 2.745249 | 2.631799 | -1.52286 |
| C | -4.65724 | -0.70629 | -2.15709 | H | 3.754851 | 2.76224 | -2.98252 |
| C | -2.95594 | 2.489649 | 0.529879 | H | 4.516202 | 2.674918 | -1.3751 |
| C | -2.12624 | 1.292708 | 0.978121 | H | 6.562765 | -0.68638 | -1.8289 |
| C | -0.71226 | 1.110367 | 0.574934 | H | 6.283329 | 0.912337 | -1.07827 |
| C | 1.432065 | 0.456147 | 1.374278 | H | 5.087552 | -0.403 | 0.53468 |
| C | 1.743851 | -1.00134 | 1.118279 | H | 4.69642 | -2.37214 | -1.77786 |
| C | 2.871087 | -1.37003 | 1.76131 | H | 3.914549 | -3.94086 | -0.1878 |
| C | 3.352821 | -0.25637 | 2.604138 | H | 3.144184 | -3.48799 | 1.987009 |
| C | 2.287679 | 0.841406 | 2.578909 | H | 4.586923 | -2.51388 | 2.198894 |
| C | 3.684288 | -2.62339 | 1.582483 | H | 1.073143 | -1.35399 | -0.86291 |
| C | 4.05627 | -2.91146 | 0.138376 | H | -0.12174 | -1.73767 | 0.378298 |
| C | 4.512358 | -2.0317 | -0.7597 | H | 1.254053 | -2.83325 | 0.103476 |
| C | 4.793558 | -0.57248 | -0.50364 | H | 1.713161 | 1.0193 | 0.483248 |
| C | 5.797534 | 0.018696 | -1.49564 | H | -2.36298 | 0.893158 | 1.958587 |
| C | 3.725691 | 0.791482 | -2.08756 | H | -3.47397 | 3.785043 | 2.171539 |
| C | 2.605574 | 0.224854 | -2.94737 | H | -4.8007 | 3.494713 | 1.03253 |
| C | 3.684298 | 2.313841 | -1.98688 | H | -4.34594 | 2.247489 | 2.20317 |
| C | 0.946523 | -1.78601 | 0.136586 | H | -1.60345 | 3.163582 | -1.03417 |
| C | -3.95211 | 3.024729 | 1.543301 | H | -1.81661 | 4.310003 | 0.300495 |
| C | -2.32864 | 3.571709 | -0.3289 | H | -3.11093 | 4.096499 | -0.88992 |
| H | -8.2929 | -3.27292 | -1.53283 | H | -2.75614 | 1.048648 | -1.10464 |
| H | -7.68969 | -3.37528 | 0.155905 | O | -6.63755 | -1.11677 | 0.968897 |
| H | -8.73124 | -2.01781 | -0.3251 | O | -6.79955 | -1.97787 | -1.11167 |
| H | -3.77388 | -0.13315 | -2.44582 | O | -0.2623 | 1.29774 | -0.5423 |
| H | -5.48087 | -0.42381 | -2.82204 | O | 0.02645 | 0.686432 | 1.627373 |
| H | -4.45454 | -1.76623 | -2.34613 | O | 4.424912 | -0.20635 | 3.184675 |
| H | -4.79678 | 0.3617 | 1.158452 | O | 4.976965 | 0.342774 | -2.61123 |
| H | 1.69058 | 0.792491 | 3.496751 | O | 3.640063 | 0.25846 | -0.75573 |

**Table S14.** Atomic coordinates (Å) of **4-2g** obtained at the CAM-B3LYP/TZVP level of theory in the MeOH.

| C | 2.305378 | 2.102839 | 0.213378 | H | -4.34077 | 3.423694 | -0.36839 |
| --- | --- | --- | --- | --- | --- | --- | --- |
| C | 2.943667 | 0.908094 | -0.34576 | H | 1.034665 | -1.43272 | -1.37213 |
| C | 3.558726 | -0.07941 | 0.333124 | H | -0.73561 | -1.30175 | -1.56062 |
| C | 3.986079 | -1.24374 | -0.49066 | H | 0.100891 | -0.34633 | -0.31923 |
| C | 4.90996 | -3.40848 | -0.44207 | H | 0.766554 | -3.36569 | 1.889336 |
| C | 3.810939 | -0.11213 | 1.816253 | H | 0.95562 | -1.60158 | 1.826106 |
| C | 2.302991 | 3.46193 | -0.45905 | H | 1.949658 | -2.63697 | 0.777628 |
| C | 1.041709 | 2.620597 | -0.50656 | H | -1.61893 | -5.08534 | -0.88468 |
| C | -0.15969 | 2.84407 | 0.331669 | H | -0.97631 | -5.0259 | 0.782497 |
| C | -2.52906 | 2.422934 | 0.362929 | H | -2.89522 | -3.64819 | 1.147421 |
| C | -2.74898 | 1.029336 | 0.933244 | H | -2.9695 | -3.33046 | -1.80302 |
| C | -3.75973 | 0.39402 | 0.303832 | H | -4.53128 | -1.52113 | -1.6484 |
| C | -4.37919 | 1.288841 | -0.70145 | H | -5.36298 | -0.9583 | 0.613496 |
| C | -3.64492 | 2.630262 | -0.66024 | H | -3.83951 | -1.46751 | 1.367082 |
| C | -4.27405 | -1.00884 | 0.476873 | H | -2.13934 | -0.47153 | 2.355665 |
| C | -3.98511 | -1.82964 | -0.76033 | H | -1.92422 | 1.225655 | 2.890582 |
| C | -3.11175 | -2.83627 | -0.84315 | H | -0.82813 | 0.51494 | 1.720295 |
| C | -2.26562 | -3.33585 | 0.307056 | H | -2.48164 | 3.185448 | 1.143055 |
| C | -1.26674 | -4.42239 | -0.09011 | H | 0.821246 | 2.15054 | -1.45897 |
| C | -0.11605 | -2.44496 | 0.150169 | H | 4.102942 | 3.812133 | -1.59879 |
| C | 0.086347 | -1.30691 | -0.84106 | H | 2.939068 | 2.773938 | -2.43578 |
| C | 0.961008 | -2.51724 | 1.226715 | H | 2.638819 | 4.512804 | -2.31114 |
| C | -1.87654 | 0.536717 | 2.038343 | H | 1.811119 | 4.529633 | 1.369316 |
| C | 3.034966 | 3.641272 | -1.77744 | H | 1.949525 | 5.554282 | -0.06764 |
| C | 2.368894 | 4.679521 | 0.444072 | H | 3.414047 | 4.903581 | 0.688126 |
| H | 5.656562 | -3.17503 | -1.20526 | H | 2.221902 | 2.153091 | 1.294779 |
| H | 5.322744 | -4.082 | 0.309238 | O | 3.847468 | -1.35445 | -1.69593 |
| H | 4.03684 | -3.85555 | -0.92458 | O | 4.532235 | -2.22193 | 0.27224 |
| H | 3.310875 | -0.96554 | 2.285804 | O | -0.18174 | 3.275298 | 1.469596 |
| H | 3.464935 | 0.801181 | 2.305116 | O | -1.25725 | 2.433593 | -0.34538 |
| H | 4.880837 | -0.22343 | 2.024277 | O | -5.32668 | 1.01511 | -1.41702 |
| H | 2.868498 | 0.76747 | -1.42065 | O | -0.17134 | -3.66974 | -0.59065 |
| H | -3.24802 | 2.883141 | -1.64696 | O | -1.38211 | -2.32589 | 0.821864 |
